# Age and frailty are independently associated with increased COVID-19 mortality and increased care needs in survivors: results of an international multi-centre study

**DOI:** 10.1093/ageing/afab026

**Published:** 2021-02-05

**Authors:** Geriatric Medicine Research Collaborative, Mustafa Alsahab, Lucy Beishon, Bryony Brown, Elinor Burn, Jenni K Burton, Natalie Cox, Melanie Dani, Muhammed Elhadi, Sarah Freshwater, Victoria Gaunt, Adam Gordon, Marie Goujon, Matthew Hale, Terry Hughes, Thomas A Jackson, Benjamin Jelley, Asma Khan, Heena Khiroya, Rajni Lal, Katy Madden, Laura Magill, Jane Masoli, Tahir Masud, Lauren McCluskey, Natalie McNeela, Awolkhier Mohammedseid-Nurhussien, Hannah Moorey, Mary Ni Lochlainn, Krishnarajah Nirantharakumar, Kelvin Okoth, Christopher N Osuafor, Katherine Patterson, Grace M E Pearson, Rita Perry, Michala Pettitt, Jennifer Pigott, Thomas Pinkney, Terence Quinn, Abigail Reynolds, Sarah Richardson, Nik Sanyal, Adam Seed, Isobel Sleeman, Chee Soo, Claire Steves, W David Strain, Joanne Taylor, Kelli Torsney, Carly Welch, Daisy Wilson, Miles Witham, Covid Collaborative, Hossam Aldein S Abd Elazeem, Mohammed H Abdelhafez, Amir Abdelmalak, Omar A Abdelwahab, Osama M A S Abdulhadi, Olubayode Adewole, Mohammed Ahmad, Eltayeb A Ahmed, Hazem Ahmed, Islam A Ahmed, Mertcan Akcay, Yeşim Akdeniz, Emrah Akın, Carolyn Akladious, Francesco Alessandri, Ali Ali, Abdulmalek Aljafari, Abdulmoiz Aljafari, Mohammed Al-Sadawi, Lobna Al-Sodani, Fatih Altintoprak, Gitanjali Amaratungaz, Jocelyn Amer, Sylvia Amini, Taha Amir, Cheran Anandarajah, Rachael Anders, Muhammed H Ansari, Kingsley Appiah, Jolene Atia, Catherine Atkin, Avinash Aujayeb, Elsayed M Awad, Mohammed A Azab, Mohammad T Azam, Sally Aziz, Ahmed Y Azzam, Laxmi Babar, Laura Babb, Manpreet Badh, Clare Baguneid, Emily Bailey, Efstratia Baili, Sarah Baldwin, Ioannis Baloyiannis, Moulinath Bannerjee, Anna Barnard, Fabio Barra, Hannah Bashir, Monica Bawor, Zülfü Bayhan, Lucy Beishon, James Belcher, Ravindra Belgamwar, Corrina Bentley, Amy Birchenough, Yen Nee J Bo, Hayley R Boden, Ahmad Bouhuwaish, Gioia Brachini, Laura Bremner, Hannah Bridgwater, Catherine Bryant, Gabrielle Budd, Sharon Budd, Adam Budzikoski, Reem Bulla, Antonio Buondonno, Antonio Buondonno, Nicole Burden, Elinor Burn, Hejab Butt, Recayi Capoglu, Andra Caracostea, Rifa Cardoso, Alexis Carr, Milagros Carrasco-Prats, Caterina Cattel, Giancarlo Ceccarelli, Giuseppe Cecere, Alexandros Charalabopoulos, Evelyn Charsley, Hannah Cheney-Lowe, Theodore Chevallier, Asad J Choudhry, Flavia Ciccarone, Pierfranco M Cicerchia, Bruno Cirillo, Fatma D Collins, Victoria Comerford, Ahmed Cordie, Siobhan Coulter, Nick Coulthard, Catrin Cox, Victoria Cox, Andrew Crowe, Jack Cullen, Jean Cummings, Niamh Cunningham, Daniel Curley, Hannah Currie, Madeleine Daly, Jay Darley, Nikhita Dattani, Spyridon Davakis, Rowan Davies, Gilda De Paola, Giorgio De Toma, Sergio Del Valle-Ruiz, Benyamin Deldar, Hakan Demir, Arjun Desai, Nirali Desai, Alice Devaney, Lindsey Dew, Jugdeep Dhesi, Maria Dias, Gordon Dick, Parveen Doddamani, Gurinder Dogra, Tina Doll, Hannah C Dooley, Samiullah Dost, Catherine Dotchin, Hannah Dowell, Ioan M Draghita, James M Dundas, Giulia Duranti, Hiren Dusara, Rajesh Dwivedi, Adam H Dyer, Alison Eastaugh, Elinor Edwards, Shrouk M Elghazaly, Ahmed O Elmehrath, Hope Elrick, Mostafa El-Shazly, Alexander Emery, Eric W Etchill, Sarah Evans, Felicity Evison, Cassandra Fairhead, Margherita Faulkner, Agnieszka Felska, Antia Fernandez, Pedro V Fernández-Fernández, Antonella Ferraiolo, Simone Ferrero, Enrico Fiori, Necattin Firat, Gracie Fisk, Anna Fleck, Giovanni B Fonsi, Alodia Gabre-Kidan, Gaetano Gallo, Ratnam Gandhi, Madeleine Garner, Nikolaos Georgiou, Hannah Gerretsen, Nourhan A A Ghannam, Andrew Ghobrial, Hedra Ghobrial, Zaynub Ghufoor, Jake Gibbon, Georgia F Gilbert, Marie Giles, Clara Giménez-Francés, Emre Gonullu, Amy Gray, Joshua H Gray, Deirdre Green, Charlotte Greene, Ellanna Griffin, Karla Griffith, Anthony Grubb, Yue Guan, Daniel N Guerero, Ayushi Gupta, Claudio Gustavino, Laurenny Guzman, Ahmed K M Hadreiez, Jiannis Hajiioannou, Deevia Hanji, Deepthy Hari Madhavan, Tarık Harmantepe, Patrick Harrison, Barbara Hart, Aidan Haslam, Victoria Haunton, Elliott R Haut, Torben Heinsohn, Lindsay Hennah, Helal F Hetta, Alexander Hickman, Abigail Hobill, Patrick C P Hogan, Vesna Hogan, Elizabeth Holmes, Katie Honney, Katharine Hood, Katherine Hopkinson, Lara Howells, Nicole Hrouda, Danielle Hunsley, William Hurst, Rand A Hussein, Mohamed Eltaher A A Ibrahim, Ishmam Ibtida, Aina Ibukunoluwakitan, Irem Ishlek, Rishi Iyer, Karl Jackson, Rosie Jackson, Ellen James, Hayley Jarvis, Sophie Jeffs, Nathan Jenko, Sasha Jeyakumar, Shahriar Kabir, Harjinder Kainth, Jason Kalloo, Akhil Kanzaria, Amalia Karapanou, Nuha Kardaman, Sandeep Karthikeyan, Anne Karunatilleke, Mairead Kelly, Nicola I Kelly, Hesham Khalid, Haris Khan, Muhammad S Khan, Matthew King, Thomas Kneen, Li Kok, Chiara Kratochwila, Aneliya Kuzeva, Pierfrancesco Lapolla, Rebecca Lau, Kar Yee Law, Aimee Leadbetter, Gabriel Lee, Helena Lee, Helena Lee, Gavriella Levinson, Grace Lewis, Theodore Liakakos, Stephen Lim, Danielle Lis, Emma Livesey, Pedro López-Morales, Lily Lowes, Eleanor Lunt, Emily Lyon, Suvira Madan, Zeinab Majid, Harsha Malapati, Jade Man, Baguiasri Mandane, Sarah H Manning, Baris Mantoglu, Nuria Martínez-Sanz, William Marx, Almontacer E B Masood, Tom Maughan, Jamie Mawhinney, Dominic Maxfield, Jordan Mayer, Henry Maynard, Claire McDonald, Aine McGovern, Sophie Mclachlan, Esther Medina-Manuel, Simona Meneghini, Michelle Metcalf, John Millwood-Hargrave, Andrea Mingoli, Kelvin Miu, Fawsiya Mohamed, Soha M Mohamed, Aliae A R Mohamed Hussein, Abdulkader Mohammad, Aaliya Mohammed, Ahmed A Momen, Farhana Moomo, Ismael Mora-Guzmán, Lizzie Moriarty, Hamilton Morrin, Claire Morris, Nicholas Moss, Mohamed M Moustafa, Maria Mpoura, Mohammed Mubin, Ali Muhtaroglu, Georgina Muir, Stephanie Mulhern, Daniel Muller, Declan C Murphy, Bushra Muzammil, Varun Nadkarni, Mariam Albatoul Nageh, Yasmin K NasrEldin, Wasim Nawaz, Hanna Nguyen, Cliona Ni Cheallaigh, Alexander Noar, Samuel North, Favour Nwolu, Alice O’Docherty, Omoteniola Odutola, Sinead O’Dwyer, Olebu Ogochukwu, Catherine O’Mahony, Lia Orlando, Marc Osterdahl, Christina Page, Ismini Panayotidis, Shivam Pancholi, Jessica Parkin, Lauren C Passby, Patricia Pastor-Pérez, Harnish Patel, Shefali Patel, Rose Penfold, Rupini Perinpanathan, Konstantinos Perivoliotis, Teresa Perra, Martha Pinkney, Enrico Pinotti, Alberto Porcu, Angeline Price, Francesco Pugliese, Prabhleen Puri, Sylvia Pytraczyk, Yusra Qaiser, Maria Qurashi, Dina Radenkovic, Thurkka Rajeswaran, Sarah F Rapaport, Tahmina Razzak, Lara Reilly, Paul Reynolds, Alexandra Richardson, Amelia Roberts, Amelia Roberts, Charlotte Roberts-Rhodes, Tanya Robinson, Aldo Rocca, Emily Ross-Skinner, Miguel Ruiz-Marín, Rebecca Ryall, Alshaimaa M Saad, Mahmoud M Saad, Ambreen Sadiq, Giuseppe Sammarco, Michail A Sampanis, Hazel Sanghvi, Paolo Sapienza, Ross Sayers, Luca Scott, Michael Sen, Mosab A A Shaban, Kathleen T Shakespeare, Ellie Shaw, Hannah Shaw, Jonathan Sheldrake, Sing Yang Sim, Luigi Simonelli, Nikolaos V Sipsas, Jarita Sivam, Sri Sivarajan, Jennifer Smith, Fabio Speranza, Claire Spice, Amanda Stafford, Katharine Stambollouian, Kent A Stevens, Jack Stewart, Emma Stratton, Hannah Street, Michael Surtees, Emma Swinnerton, Ahmed S A Taher, Caroline Tait, Amybel Taylor, Miriam Thake, Katie Thin, Hannah Thould, Thyn Thyn, Benjaman To, Hannah Tobiss, Kathryn Toppley, Liam Townsend, Ellen Tullo, George Tzovaras, Anthony Umeadi, Hrisheekesh Vaidya, María Valero-Soriano, Rosanna Varden, Vittoria Vergani, Dominique Vervoort, Giuseppina Vescio, Mark Vettasseri, Madiha Virk, Vaishali Vyas, Joanne Wagland, Stephanie Wallis, Chloe Warner, Eleanor Watkins, Hannah Watson, Rachael Webb, Sarah H Welsh, Ruth West, Elisha Whelan, Julie Whitney, Mark Whitsey, Catherine Wilcock, Iain Wilkinson, David Williams, Megan Williamson, Ruth H Willott, Mettha Wimalasundera, Yu Lelt Win, Laura Winter, Stephanie Worrall, Rebecca Wright, Natalie Yeo, Eirene Yeung, Merve Yigit, Yasin A Yildiz, Humza Yusuf, Martina Zambon, Hein Zaw, Omar Zein Elabedeen, Carly Welch

**Keywords:** frailty, COVID-19, mortality, transitions of care, delirium

## Abstract

**Introduction:**

Increased mortality has been demonstrated in older adults with coronavirus disease 2019 (COVID-19), but the effect of frailty has been unclear.

**Methods:**

This multi-centre cohort study involved patients aged 18 years and older hospitalised with COVID-19, using routinely collected data. We used Cox regression analysis to assess the impact of age, frailty and delirium on the risk of inpatient mortality, adjusting for sex, illness severity, inflammation and co-morbidities. We used ordinal logistic regression analysis to assess the impact of age, Clinical Frailty Scale (CFS) and delirium on risk of increased care requirements on discharge, adjusting for the same variables.

**Results:**

Data from 5,711 patients from 55 hospitals in 12 countries were included (median age 74, interquartile range [IQR] 54–83; 55.2% male). The risk of death increased independently with increasing age (>80 versus 18–49: hazard ratio [HR] 3.57, confidence interval [CI] 2.54–5.02), frailty (CFS 8 versus 1–3: HR 3.03, CI 2.29–4.00) inflammation, renal disease, cardiovascular disease and cancer, but not delirium. Age, frailty (CFS 7 versus 1–3: odds ratio 7.00, CI 5.27–9.32), delirium, dementia and mental health diagnoses were all associated with increased risk of higher care needs on discharge. The likelihood of adverse outcomes increased across all grades of CFS from 4 to 9.

**Conclusion:**

Age and frailty are independently associated with adverse outcomes in COVID-19. Risk of increased care needs was also increased in survivors of COVID-19 with frailty or older age.

## Key points

- Age and frailty were independently associated with increased risk of mortality in hospitalised patients with COVID-19.
- Delirium was not predictive of mortality but was predictive of critical care admission with COVID-19.
- Age, frailty and delirium were associated with increased odds of transitions of care needs at discharge in survivors.

## Background

Coronavirus disease 2019 (COVID-19) is a multi-system disease caused by the severe acute respiratory syndrome coronavirus 2 (SARS-CoV-2). Early data suggested older and/or co-morbid adults were at increased risk of adverse outcomes (1–4). The clinical frailty scale (CFS) (5, 6) featured in critical care escalation guidelines in UK and Canada (7). Early evidence of impact of frailty produced mixed findings; studies suggested frailty was (8), and was not (9, 10) associated with increased mortality with COVID-19. Our collaborative previously demonstrated in other conditions that delirium is common and associated with adverse outcomes, especially in frail older adults (11). Two distinct cohorts identified that delirium is more common in frail older adults with COVID-19 (12). Thus, a need for research on delirium and frailty with COVID-19 was recognised (13). Additionally, prolonged recovery has been reported in survivors (14), although the impact of COVID-19 upon transitions of care needs with age and frailty has not been previously studied.

## Objective

To evaluate the association of age, frailty and delirium with adverse outcomes including mortality and secondary outcomes to include critical care admission, incident delirium and transitions of care needs in survivors in hospitalised patients with COVID-19.

## Methods

### Study design and setting

We included unscheduled hospital admissions of adults aged ≥ 18 years old with COVID-19 infection in this observational study. Emergency department discharges and nosocomial COVID-19 were excluded. Prospective data upload upon clinical suspicion was encouraged; clinicians identified patients during medical clerking, or ward transfer. Retrospective identification was dependent on local COVID-19 coding processes, involving medical records, informatics or microbiology.

Investigation was led through the Geriatric Medicine Research Collaborative (GeMRC) (15, 16). The protocol was openly available on GeMRC, British Geriatrics Society and University of Birmingham REDCap webpages, and disseminated via emails and social media. Sites were required to obtain local, regional and national approvals, and declare these were in place when registering. Data sharing agreements were arranged where required. Sites were provided REDCap data upload logins; secure encrypted web-based data management software. Sites uploaded anonymised patient-level data onto REDCap. Independent data managers ensured quality control.

### Case definition and laboratory confirmation

Suspected COVID-19 infection was diagnosed clinically considering symptoms, radiology and laboratory tests. Laboratory confirmation was conducted according to local policies and World Health Organization guidance; identification of SARS-CoV-2 from reverse transciptase polymerase chain reaction from oropharyngeal or high nasal swabs (RdRp gene assay), or antibodies against SARS-CoV-2 in serum samples ≥ 14 days after symptom onset. Patients were included if there was strong clinical suspicion but no laboratory confirmation.

### Variables and data sources

Data were extracted from routinely collected clinical information; variables are outlined in the online supplement (Supplementary Table S1). Screening with the 4 ‘A’s Test (4AT) (17) on admission was recommended; ≥4 was suggestive of prevalent delirium. Incident delirium was defined as documented emergent delirium during admission. Frailty was derived based on function 2 weeks before admission using the 9-point CFS (6), by prospective clinical assessment or retrospectively from medical records.

### Study outcomes

Primary end point was death during index admission. Secondary end points were critical care admission, incident delirium and increased care requirements at discharge (as defined below).

### Statistical methods

Data analysis was performed using STATA SE version 16 (StataCorp LLC, Texas, USA) by an independent statistician (KO). Descriptive variables were expressed as median and interquartile range (IQR), and counts; chi-squared and Mann–Whitney U tests were applied for statistical significance of mortality differences.

### Primary outcome

We used Cox proportional survival analysis to assess impact of delirium and frailty upon inpatient mortality. Univariable and multivariable analyses were conducted as follows:

- Model 1: variables previously associated with COVID-19 adverse outcomes; age (3, 18), sex (3, 18), C-reactive protein (CRP) (3), Ferritin (19, 20), body mass index (BMI) (21), Alanine aminotransferase (3), lymphocyte: neutrophil ratio, Glomerular Filtration Rate [Modification of Diet in Renal Disease, MDRD, formula (22)] (3), co-morbidities coded individually (3, 18) and illness severity (3) by admission national early warning score (NEWS) (23).
- Model 2: variables above with CFS; CFS 1–3 was the comparison group, and CFS 9 and missing CFS were separate discrete groups (6). Additional analyses were conducted with age and frailty (excluding CFS 9) as continuous variables.
- Model 3: variables in Models 1 and 2 and delirium (prevalent/incident).

We performed Wald and Likelihood Ratio tests for model fit for age and frailty individually and together as predictors in all models. In addition, we assessed for multiplicative interactions between age and frailty upon the primary outcome of mortality censored at the point of discharge. Frailty was grouped with the exclusion of CFS 9 in these models (CFS 1–3, CFS 4–6, CFS 7–8, CFS missing).

### Sensitivity analysis

We performed sensitivity analyses on models for the primary outcome:

1. Excluding patients aged < 65 years old.
2. Excluding patients admitted to hospitals outside the UK.

### Secondary outcomes

We used binary logistic regression to assess impact of variables on critical care admission and incident delirium (excluding prevalent delirium), and ordinal logistic regression to assess impact upon care requirements at discharge. Incident delirium was considered as any new diagnosis of delirium by a healthcare professional at any time during admission, where this was not present at admission. Increased care was defined as transitions across three care levels: living at home without formal care, living at home with formal care or living in a 24-h long-term care facility.

## Results

The study includes data from 5,711 individuals with COVID-19 admitted to 55 hospitals in 12 countries. Supplementary Figure S1 shows reasons for data exclusion. Supplementary Table S2 demonstrates participating site locations. Median age was 74 and 55.2% were male. Table 1 shows full baseline patient characteristics.

**Table 1:** Baseline characteristics of patients included in study

		All patients	Death during admission		
		(*N* = 5,711)	Yes (*N* = 1,596)	No (*N* = 4,115)	*P* value
Age	Median (IQR) (*N* = 5,711)	74 (58–83)	80 (72–87)	69 (54–82)	<0.001
	Distribution—*N*(%)				<0.001
	18–49	817 (14.3)	49 (3.1)	768 (18.7)	
	50–64	1,118 (19.6)	156 (9.8)	962 (23.4)	
	65–79	1,698 (29.7)	537 (33.7)	1,161 (28.2)	
	≥80	2,078 (36.4)	854 (53.5)	1,224 (29.7)	
Female—*N* (%)		2,562 (44.9)	624 (39.1)	1,938 (47.1)	<0.001
Temperature: distribution—*N*(%)	<36°C	391 (6.9)	134 (8.4)	257 (6.3)	0.001
	36.0–37.5°C	2,977 (52.1)	776 (48.6)	2,201 (53.5)	
	37.5–37.9°C	699 (12.2)	223 (14.0)	476 (11.6)	
	38.0–39.0°C	1,271 (22.3)	348 (21.8)	923 (22.4)	
	>39.0°C	260 (4.6)	83 (5.2)	177 (4.3)	
	Missing	113 (2.0)	32 (2.0)	81 (2.0)	
Oxygen requirement: distribution—*N* (%)	None (FiO_2_ 21%)	2,215 (38.8)	574 (36.0)	1,641 (39.9)	<0.001
	FiO_2_ 22–29%	423 (7.4)	96 (6.0)	327 (8.0)	
	FiO_2_ 30–39%	227 (4.0)	66 (4.1)	161 (4.0)	
	FiO_2_ ≥ 40%	864 (15.1)	392 (24.6)	484 (11.5)	
	Missing	1,982 (34.7)	468 (29.3)	1,514 (36.8)	
Body mass index	Median (IQR) (*N* = 3,599)	26.7 (23.1–31.0)	26.0 (22.4–30.5)	26.9 (23.4–31.2)	0.002
	Distribution—*N*(%)				<0.001
	<18.5	163 (2.9)	46 (2.9)	117(2.8)	
	18.5–24.9	1,221 (21.4)	333 (20.9)	888 (21.6)	
	25.0–29.9	1,123 (19.7)	234 (14.7)	889 (21.6)	
	≥30.0	1,092 (19.1)	233 (14.6)	859 (20.9)	
	Missing	2,112 (37.0)	750 (47.0)	1,362 (33.1)	
Symptoms—*N* (%)	Fever	2,997 (52.5)	783 (49.1)	2,214 (53.8)	<0.001
	Cough/breathlessness	3,976 (69.6)	1,103 (69.1)	2,873 (69.8)	0.602
	Confusion	1,161 (20.3)	444 (27.8)	717 (17.4)	<0.001
	Other	2,462 (43.1)	617 (38.7)	1,845 (44.8)	
Prevalent delirium—*N* (%)	No	4,288 (75.1)	1,087 (68.1)	3,201 (77.8)	0.001
	Yes	1,120 (19.6)	443 (27.8)	677 (16.5)	
	Missing	303 (5.3)	66 (4.1)	237 (5.8)	
Composite delirium (incident/prevalent)—*N* (%)	No	3,512 (61.5)	818 (51.3)	2,694 (65.5)	<0.001
	Yes	1,559 (27.3)	630 (39.5)	929 (22.6)	
	Missing	640 (11.2)	148 (9.3)	492 (12.0)	
Clinical frailty scale—*N* (%)	1–3	2,069 (36.2)	251 (15.7)	1,818 (44.2)	<0.001
	4	571 (10.0)	174 (10.9)	397 (9.7)	
	5	604 (10.6)	207 (13.0)	397 (9.7)	
	6	880 (15.4)	318 (19.9)	562 (13.7)	
	7	761 (13.3)	308 (19.3)	453 (11.0)	
	8	165 (3.0)	92 (5.8)	73 (1.8)	
	9	31 (0.5)	18 (1.1)	13 (0.3)	
	Missing	630 (11.0)	228 (14.3)	402 (9.8)	
Co-existing condition—*N* (%)	Any	4,765 (83.4)	1,483 (92.9)	3,282 (79.8)	<0.001
	Diabetes mellitus	1,669 (29.2)	544 (34.1)	1,125 (27.3)	<0.001
	Cardiovascular disease	2,847 (49.9)	1,013 (63.5)	1,834 (44.6)	<0.001
	Respiratory disease	1,459 (25.6)	427 (26.8)	1,032 (25.1)	0.193
	Cancer	622 (11.0)	234 (14.7)	388 (9.4)	<0.001
	Mental health	482 (8.4)	124 (7.8)	358 (8.7)	0.256
	Dementia	911 (16.0)	387 (24.3)	524 (12.7)	<0.001
	Human immunodeficiency virus	16 (0.3)	0 (0.0)	16 (0.4)	0.013
Previous residence—*N* (%)	Own home no formal care	3,453 (60.5)	760 (47.6)	2,693 (65.4)	<0.001
	Own home with formal care	802 (14.0)	285 (17.9)	517 (12.6)	
	24-h long-term care facility	1,010 (17.7)	442 (27.7)	568 (13.8)	
	Missing	446 (7.8)	109 (6.8)	337 (8.2)	
Medications—*N* (%)	ACE-inhibitors or Angiotensin receptor blockers	1,330 (23.3)	405 (25.4)	925 (22.5)	0.001
	Non-steroidal anti-inflammatory drugs	328 (5.7)	98 (6.1)	230 (5.6)	0.003
	Steroids	509 (8.9)	163 (10.2)	346 (8.4)	<0.001
	Immunosuppressants	177 (3.1)	37 (2.3)	140 (3.4)	0.010
	Chemotherapy	86 (1.5)	18 (1.1)	68 (1.7)	0.001
	Anti-retrovirals	34 (0.6)	6 (0.4)	28 (0.7)	0.036
Neutrophil to lymphocyte ratio	Median (IQR) (*N* = 5,255)	6.0 (3.5–10.7)	8.2 (4.7–8.2)	5.4 (3.2–9.2)	<0.001
C-reactive protein	Median (IQR)—mg/l (*N* = 5,289)	76 (29–148)	111 (54–197)	63 (23–126)	<0.001
	Distribution—*N* (%)				<0.001
	<10	578 (10.1)	66 (4.1)	512 (12.4)	
	10–40	1,072 (18.8)	207 (13.0)	865 (21.0)	
	>40	3,639 (63.7)	1,199 (75.1)	2,440 (59.3)	
	Missing	422 (7.4)	124 (7.8)	298 (7.2)	
Ferritin	Median (IQR)—mg/l (*N* = 1,734)	580 (257–1,249)	681 (322–1,415)	544 (231–1,192)	<0.001
	Distribution—*N* (%)				<0.001
	<100	160 (2.8)	19 (1.2)	141 (3.4)	
	100–1,000	1,039 (18.2)	268 (16.8)	771 (18.7)	
	>1,000	535 (9.4)	149 (9.3)	386 (9.4)	
	Missing	3,977 (70.0)	1,160 (72.7)	2,817 (68.5)	
Glomerular filtration rate	Median (IQR) (*N* = 5,275)	57.6 (37.8–78.8)	43.2 (26.4–64.4)	62.7 (44.2–82.5)	0.001
	>90	851 (14.9)	147 (9.2)	704 (17.1)	<0.001
	60–89	1,611 (28.2)	291 (18.2)	1,320 (32.1)	
	45–59	1,033 (18.1)	266 (16.7)	767 (18.6)	
	30–44	877 (15.4)	345 (21.6)	532 (12.9)	
	15–29	561 (9.8)	281 (17.6)	280 (6.8)	
	<15	342 (6.0)	161 (10.1)	181 (4.4)	
	Missing	436 (7.6)	105 (6.6)	331 (8.0)	
Alanine aminotransferase (ALT)—U/l	Median (IQR) (*N* = 4,631)	24 (16–41)	24 (16–40)	24 (16–41)	0.895
	Distribution—*N* (%)				0.329
	<40	3,468 (60.7)	987 (61.8)	2,481 (60.3)	
	≥ 40	1,164 (20.4)	327 (20.5)	837 (20.3)	
	Missing	1,079 (18.9)	282 (17.7)	797 (19.4)	
Confirmation—*N* (%)	Clinical suspicion	498 (8.7)	80 (5.1)	418 (10.2)	<0.001
	PCR	5,200 (91.1)	1,514 (94.9)	3,686 (89.6)	
	Antibody test	13 (0.2)	2 (0.1)	11 (0.3)	
Outcomes					
Length of stay/days to death—median (IQR) (*N* = 4,939)		8 (4–16)	7(4–13)	9 (4–18)	
Incident delirium—*N* (%)	None	3,985 (69.8)	957 (60.0)	3,028 (73.6)	<0.001
	Incident with no documented prevalent delirium	439 (7.7)	187 (11.7)	252 (6.1)	
	Incident delirium with documented prevalent delirium	748 (13.1)	321 (20.1)	427 (10.4)	
	Missing	539 (9.4)	9.44 (8.2)	408 (10.0)	
Critical care admission—*N* (%)	No	5,063 (86.7)	1,370 (85.8)	3,693 (89.7)	<0.001
	Yes	647 (11.3)	226 (14.2)	421 (10.2)	
	Missing	1 (<0.1)	0 (0.0)	1 (<0.1)	

ACE, angiotensin-converting enzyme inhibitors; FiO_2_, fraction of inspired oxygen; PCR, polymerase chain reaction; *P*-values, chi-squared tests for categorical data, and Mann–Whitney U-test for continuous data.

### Mortality

**Figure 1:**
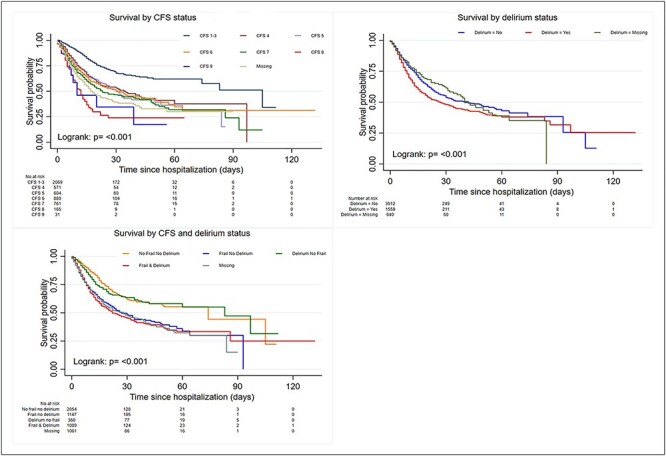
Kaplan–Meier curves demonstrating risk of death with frailty and delirium, An increased risk of death was demonstrated in the most severely frail patients, whereas delirium was not associated with risk of death in this cohort.

Risk of death increased independently with age and frailty in univariable and multivariable analyses (Table 2), including with age and frailty as continuous variables (Supplementary Table S3, online supplement). Risk of death tripled >80 years old (hazard ratio [HR] 3.57, 95% confidence interval [CI] 2.54–5.02), compared with 18–50, and in very severely frail individuals (CFS 8 versus CFS 1–3) (HR 3.03, 95% CI 2.29–4.00). Models 1 and 2 are available online (Supplementary Table S4). Age and frailty together and not individually as predictor variables improved model of fit (LR χ^2^ (7) = 91.3, *P* < 0.001; Wald χ^2^ (10) = 207.9, *P* < 0.001). Additionally, mortality risk increased with age and frailty together in multiplicative interactions (Supplementary Table S5 and Supplementary Figure S2). Delirium was predictive of mortality in univariable but not multivariable analysis. Risk of death increased with higher CRP or ferritin, more severe renal disease and cancer. Mortality did not differ across BMI cut-offs; risk of death was increased with missing BMI. Figure 1 demonstrates Kaplan–Meier curves for risk of death for frailty and delirium. Results were not affected by sensitivity analyses for ≥65 years old, or UK data only (Supplementary Tables S6 and S7).

**Table 2:** Cox regression models for risk of death

	Univariable			Multivariable		
	HR	95% CI	*P*-value	HR	95% CI	*P*-value
Delirium						
No	Ref			Ref		
Yes	1.30	1.17–1.44	<0.001	0.97	0.86–1.09	0.588
Missing	0.95	0.80–1.13	0.562	0.79	0.65–0.97	0.028
Frailty distribution						
1–3	Ref			Ref		
4	2.08	1.71–2.52	<0.001	1.63	1.32–2.02	<0.001
5	2.30	1.91–2.76	<0.001	1.68	1.36–2.08	<0.001
6	2.32	1.96–2.74	<0.001	1.77	1.45–2.17	<0.001
7	2.62	2.22–3.10	<0.001	1.90	1.54–2.34	<0.001
8	4.48	3.53–5.69	<0.001	3.03	2.29–4.00	<0.001
9	4.15	2.57–6.70	<0.001	2.37	1.38–4.06	0.002
Missing	3.15	2.63–3.77	<0.001	2.42	1.96–2.99	<0.001
Age distribution						
18–49 years	Ref			Ref		
50–64 years	2.03	1.47–2.80	<0.001	1.96	1.38–2.77	<0.001
65–80 years	4.04	3.01–5.41	<0.001	2.93	2.10–4.09	<0.001
>80 years	5.07	3.80–6.77	<0.001	3.57	2.54–5.02	<0.001
Sex						
Female	Ref			Ref		
Male	1.29	1.17–1.43	<0.001	1.22	1.09–1.36	0.001
NEWS						
0–4 (Low risk)	Ref			Ref		
5–6 (Medium risk)	1.43	1.24–1.65	<0.001	1.53	1.31–1.78	<0.001
≥7 (High risk)	2.14	1.90–2.41	<0.001	2.11	1.85–2.41	<0.001
Missing	2.00	1.71–2.35	<0.001	1.75	1.47–2.09	<0.001
CRP						
<10 mg/l	Ref			Ref		
10–40 mg/l	1.48	1.12–1.95	0.006	1.23	0.92–1.65	0.157
>40 mg/l	2.53	1.98–3.24	<0.001	1.87	1.44–2.44	<0.001
Missing	2.55	1.89–3.44	<0.001	2.22	1.55–3.18	<0.001
Ferritin						
<100 ng/ml	Ref			Ref		
100–1,000 ng/ml	2.05	1.29–3.27	0.002	1.83	1.14–2.93	0.012
>1,000 ng/ml	1.95	1.21–3.15	0.006	1.75	1.07–2.85	0.025
Missing	2.32	1.47–3.64	<0.001	1.90	1.20–3.00	0.006
Alanine transferase						
<40 IU/l	Ref			Ref		
>40 IU/l	1.04	0.92–1.18	0.557	1.16	1.01–1.33	0.033
Missing	1.11	0.97–1.27	0.120	0.95	0.81–1.11	0.502
Neutrophil: lymphocyte ratio	1.01	1.01–1.01	<0.001	1.00	1.00–1.01	0.018
BMI						
18.5–25 kg/m^2^	Ref			Ref		
<18.5 kg/m^2^	0.87	0.64–1.18	0.371	0.73	0.52–1.02	0.069
25–30 kg/m^2^	0.82	0.69–0.97	0.018	0.95	0.79–1.13	0.539
>30 kg/m^2^	0.79	0.67–0.93	0.006	1.03	0.86–1.24	0.758
Missing	1.40	1.23–1.59	<0.001	1.43	1.24–1.64	<0.001
eGFR (ml/min/1.73 m^2^)						
>90	Ref			Ref		
60–89	0.93	0.76–1.14	0.479	0.75	0.61–0.92	0.006
45–59	1.27	1.04–1.55	0.022	0.93	0.75–1.15	0.485
30–44	1.95	1.61–2.37	<0.001	1.22	0.99–1.50	0.056
15–29	2.50	2.05–3.06	<0.001	1.37	1.10–1.70	0.004
<15	2.37	1.89–2.96	<0.001	1.51	1.19–1.93	0.001
Missing	0.94	0.73–1.21	0.632	1.01	0.68–1.50	0.966
Comorbidities						
Diabetes mellitus	1.22	1.10–1.36	<0.001	1.07	0.96–1.20	0.231
Cardiovascular disease	1.60	1.45–1.77	<0.001	1.08	0.96–1.21	0.199
Respiratory disease	1.07	0.96–1.20	0.231	0.94	0.84–1.06	0.320
Cancer	1.33	1.16–1.53	<0.001	1.20	1.04–1.39	0.015
Mental health	0.94	0.78–1.13	0.495	0.86	0.70–1.04	0.119
Dementia	1.42	1.27–1.60	<0.001	1.06	0.92–1.22	0.400

eGFR, Estimated Glomerular Filtration Rate by Modified Diet in Renal Disease formula.

### Critical care admission

Critical care admissions were more likely with greater illness severity, CRP, or ferritin or BMI < 18.5 or ≥30 (Table 3), and less likely with age, frailty and dementia. Admissions were six times less likely in >80 years old compared with 18–50 (odds ratio [OR] 0.13, 95% CI 0.08–0.21). Delirium was independently associated with critical care admission (OR 2.67, 95% CI 2.06–3.46). Models 1 and 2 are available online (Supplementary Table S8).

**Table 3:** Odds ratios derived from logistic regression for secondary outcome of critical care admission

	Univariable			Multivariable		
	OR	95% CI	*P*-value	OR	95% CI	*P*-value
Delirium						
No	Ref			Ref		
Yes	1.01	0.84–1.22	0.886	2.67	2.06–3.46	<0.001
Missing	1.01	0.78–1.32	0.925	1.10	0.79–1.54	0.566
Frailty distribution						
1–3	Ref			Ref		
4	0.54	0.41–0.71	<0.001	0.73	0.52–1.01	0.059
5	0.27	0.19–0.37	<0.001	0.37	0.24–0.56	<0.001
6	0.16	0.11–0.22	<0.001	0.32	0.21–0.51	<0.001
7	0.05	0.03–0.10	<0.001	0.10	0.05–0.20	<0.001
8	0.02	0.00–0.16	<0.001	0.05	0.01–0.41	0.005
9	0.12	0.02–0.92	0.041	-	-	-
Missing	0.33	0.24–0.45	<0.001	0.54	0.37–0.79	0.002
Age distribution						
18–49 years	Ref			Ref		
50–64 years	1.15	0.92–1.44	0.227	1.02	0.78–1.34	0.868
65–80 years	0.57	0.45–0.71	<0.001	0.68	0.50–0.92	0.013
>80 years	0.07	0.05–0.11	<0.001	0.13	0.08–0.21	<0.001
Sex						
Female	Ref			Ref		
Male	1.83	1.54–2.18	<0.001	1.22	0.97–1.52	0.083
NEWS						
0–4 (Low risk)	Ref			Ref		
5–6 (Medium risk)	2.38	1.86–3.04	<0.001	1.93	1.46–2.54	<0.001
≥7 (High risk)	4.27	3.46–5.27	<0.001	4.01	3.13–5.15	<0.001
Missing	2.55	1.90–3.44	<0.001	3.42	2.36–4.96	<0.001
CRP						
<10 mg/l	Ref			Ref		
10–40 mg/l	1.60	1.02–2.51	0.040	1.44	0.87–2.40	0.154
>40 mg/l	3.17	2.13–4.72	<0.001	1.82	1.14–2.88	0.011
Missing	2.93	1.81–4.75	<0.001	3.52	1.90–6.52	<0.001
Ferritin						
<100 ng/ml	Ref			Ref		
100–1,000 ng/ml	3.54	1.77–7.07	<0.001	2.05	0.96–4.40	0.064
>1,000 ng/ml	8.72	4.35–17.49	<0.001	3.83	1.76–8.35	0.001
Missing	1.24	0.63–2.46	0.535	0.91	0.43–1.93	0.802
Alanine transferase						
<40 IU/l	Ref			Ref		
>40 IU/l	2.47	2.06–2.96	<0.001	1.18	0.94–1.48	0.144
Missing	0.73	0.56–0.95	0.017	0.70	0.50–0.98	0.039
Neutrophil: lymphocyte ratio	1.01	1.00–1.01	0.051	1.01	1.00–1.02	0.114
BMI						
18.5–25 kg/m^2^	Ref			Ref		
<18.5 kg/m^2^	0.21	0.06–0.66	0.008	0.20	0.05–0.86	0.031
25–30 kg/m^2^	1.97	1.52–2.56	<0.001	1.33	0.98–1.82	0.068
>30 kg/m^2^	2.71	2.10–3.48	<0.001	1.46	1.08–1.98	0.014
Missing	0.87	0.67–1.13	0.292	0.80	0.58–1.09	0.154
eGFR (ml/min/1.73 m^2^)						
>90	Ref			Ref		
60–89	0.82	0.64–1.05	0.11	0.90	0.67–1.22	0.504
45–59	0.85	0.65–1.11	0.226	1.28	0.91–1.79	0.160
30–44	0.71	0.53–0.95	0.022	1.21	0.83–1.77	0.322
15–29	0.76	0.55–1.06	0.108	1.34	0.87–2.07	0.184
<15	0.70	0.47–1.05	0.082	0.99	0.60–1.64	0.962
Missing	0.39	0.25–0.61	<0.001	1.01	0.55–1.86	0.973
Comorbidities						
Diabetes mellitus	1.16	0.97–1.38	0.101	1.15	0.92–1.43	0.226
Cardiovascular disease	0.74	0.63–0.87	<0.001	1.17	0.94–1.47	0.161
Respiratory disease	0.90	0.74–1.09	0.279	1.06	0.84–1.34	0.626
Cancer	0.80	0.61–1.06	0.125	1.14	0.81–1.60	0.464
Mental health	0.77	0.56–1.06	0.112	0.96	0.66–1.40	0.842
Dementia	0.06	0.03–0.12	<0.001	0.26	0.12–0.56	0.001

eGFR, Estimated Glomerular Filtration Rate by Modified Diet in Renal Disease formula.

### Incident delirium

Delirium incidence was 9.6%. Incident delirium odds increased with age but not frailty (Table 4). Risk in >80-year olds was double that of 18–50 (OR 2.21, 95% CI 1.37–3.59). Incident delirium odds were independently associated with male sex, illness severity and cardiovascular disease. Dementia was not associated with incident delirium. Model 1 is available online (Supplementary Table S9).

**Table 4:** Odds ratios derived from logistic regression for secondary outcomes of incident delirium

	Univariable			Multivariable		
	OR	95% CI	*P*-value	OR	95% CI	*P*-value
Frailty distribution						
1–3	Ref			Ref		
4	1.49	1.04–2.12	0.028	1.07	0.72–1.60	0.745
5	1.43	1.01–2.04	0.045	1.06	0.71–1.58	0.779
6	1.86	1.39–2.49	<0.001	1.24	0.86–1.79	0.258
7	1.97	1.46–2.66	<0.001	1.38	0.94–2.03	0.102
8	2.48	1.51–4.06	<0.001	1.35	0.75–2.44	0.317
9	1.17	0.28–4.97	0.830	0.41	0.05–3.10	0.386
Missing	1.21	0.84–1.75	0.299	0.96	0.63–1.45	0.831
Age distribution						
18–49 years	Ref			Ref		
50–64 years	1.57	1.01–2.42	0.045	1.29	0.82–2.03	0.272
65–80 years	2.07	1.38–3.09	<0.001	1.59	1.01–2.51	0.045
>80 years	2.93	1.99–4.30	<0.001	2.21	1.37–3.59	0.001
Sex						
Female	Ref			Ref		
Male	1.24	1.01–1.51	0.037	1.26	1.01–1.57	0.039
NEWS						
0–4 (Low risk)	Ref			Ref		
5–6 (Medium risk)	1.08	0.81–1.43	0.600	1.02	0.76–1.38	0.876
≥7 (High risk)	1.76	1.40–2.21	<0.001	1.52	1.18–1.96	0.001
Missing	1.40	1.00–1.97	0.050	1.32	0.91–1.91	0.141
CRP						
<10 mg/l	Ref			Ref		
10–40 mg/l	1.40	0.93–2.13	0.110	1.21	0.78–1.89	0.392
>40 mg/l	1.49	1.03–2.17	0.034	1.15	0.77–1.72	0.505
Missing	0.82	0.46–1.45	0.499	0.96	0.47–1.98	0.923
Ferritin						
<100 ng/ml	Ref			Ref		
100–1,000 ng/ml	1.40	0.93–2.13	0.110	1.46	0.73–2.92	0.283
>1,000 ng/ml	1.49	1.03–2.17	0.034	1.62	0.78–3.36	0.197
Missing	0.82	0.46–1.45	0.499	1.02	0.52–2.00	0.945
Alanine transferase						
<40 IU/l	Ref			Ref		
>40 IU/l	1.08	0.85–1.37	0.552	1.17	0.90–1.52	0.248
Missing	0.79	0.60–1.04	0.088	0.98	0.72–1.32	0.873
Neutrophil: lymphocyte ratio	1.01	1.00–1.02	0.002	1.01	1.00–1.01	0.175
BMI						
18.5–25 kg/m^2^	Ref			Ref		
<18.5 kg/m^2^	1.46	0.85–2.53	0.172	1.25	0.70–2.22	0.455
25–30 kg/m^2^	0.91	0.66–1.25	0.570	0.94	0.67–1.31	0.695
>30 kg/m^2^	0.95	0.70–1.31	0.767	1.15	0.82–1.62	0.423
Missing	1.16	0.89–1.51	0.260	1.06	0.80–1.40	0.688
eGFR (ml/min/1.73 m^2^)						
>90	Ref			Ref		
60–89	0.84	0.61–1.16	0.291	0.75	0.53–1.05	0.089
45–59	0.94	0.66–1.33	0.706	0.71	0.49–1.03	0.074
30–44	1.39	0.99–1.94	0.055	0.90	0.62–1.30	0.575
15–29	1.50	1.04–2.17	0.031	0.88	0.58–1.32	0.535
<15	1.14	0.72–1.80	0.577	0.76	0.46–1.27	0.299
Missing	0.59	0.35–0.99	0.046	0.58	0.25–1.35	0.210
Comorbidities						
Diabetes mellitus	1.20	0.97–1.48	0.087	1.04	0.82–1.30	0.763
Cardiovascular disease	1.67	1.37–2.04	<0.001	1.29	1.03–1.62	0.028
Respiratory disease	1.67	1.37–2.04	<0.001	1.13	0.90–1.42	0.278
Cancer	1.00	0.74–1.37	0.976	0.89	0.64–1.24	0.492
Mental health	1.00	0.70–1.42	0.993	1.07	0.75–1.55	0.701
Dementia	1.44	1.13–1.84	0.003	1.12	0.83–1.49	0.460

eGFR, Estimated Glomerular Filtration Rate by Modified Diet in Renal Disease formula.

### Transitions of care needs

Increased care risk increased with age, frailty, delirium, dementia and mental health problems (Table 5). Likelihood of increased care >80 years old was triple that for 18–50 (OR 3.07, 95% CI 2.25–4.20). Increased care levels were seven times more likely with severe frailty (CFS 7) than without frailty (CFS 1–3) (OR 7.00, 95%CI 5.27–9.32). Models 1 and 2 are available online (Supplementary Table S10).

**Table 5:** Odds ratios derived from ordinal logistic regression for secondary outcomes of increased care level on discharge

	Univariable			Multivariable		
	OR	95% CI	*P*-value	OR	95% CI	*P*-value
Delirium						
No	Ref			Ref		
Yes	4.22	3.65–4.89	<0.001	1.83	1.53–2.19	<0.001
Missing	53.46	41.80–68.37	<0.001	100.10	73.02–137.21	<0.001
Frailty distribution						
1–3	Ref			Ref		
4	2.69	2.13–3.40	<0.001	1.93	1.47–2.54	<0.001
5	3.77	3.03–4.70	<0.001	2.55	1.94–3.34	<0.001
6	8.26	6.82–10.01	<0.001	5.09	3.92–6.60	<0.001
7	10.9	8.93–13.29	<0.001	7.00	5.27–9.32	<0.001
8	10.87	7.40–15.97	<0.001	6.06	3.73–9.85	<0.001
9	7.59	2.94–19.55	<0.001	3.68	1.19–11.39	0.024
Missing	4.49	3.55–5.68	<0.001	1.36	1.00–1.85	0.049
Age distribution						
18–49 years	Ref			Ref		
50–64 years	1.56	1.24–1.97	<0.001	1.37	1.04–1.82	0.026
65–80 years	3.95	3.19–4.90	<0.001	2.20	1.66–2.93	<0.001
>80 years	7.16	5.80–8.83	<0.001	3.07	2.25–4.20	<0.001
Sex						
Female	Ref			Ref		
Male	0.70	0.62–0.79	<0.001	0.98	0.84–1.15	0.812
NEWS						
0–4 (Low risk)	Ref			Ref		
5–6 (Medium risk)	0.84	0.72–0.99	0.040	1.10	0.90–1.33	0.355
≥7 (High risk)	1.02	0.88–1.19	0.788	1.15	0.95–1.39	0.159
Missing	0.87	0.69–1.10	0.242	0.25	0.18–0.36	<0.001
CRP						
<10 mg/l	Ref			Ref		
10–40 mg/l	1.23	0.99–1.53	0.063	1.02	0.79–1.33	0.858
>40 mg/l	1.21	1.00–1.46	0.052	1.08	0.85–1.37	0.529
Missing	2.74	2.07–3.61	<0.001	3.58	2.40–5.33	<0.001
Ferritin						
<100 ng/ml	Ref			Ref		
100–1,000 ng/ml	0.88	0.62–1.24	0.460	1.07	0.71–1.62	0.745
>1,000 ng/ml	0.65	0.45–0.96	0.029	1.00	0.63–1.59	1.000
Missing	0.99	0.72–1.37	0.970	0.86	0.58–1.27	0.441
Alanine transferase						
<40 IU/l	Ref			Ref		
>40 IU/l	0.53	0.45–0.63	<0.001	0.97	0.79–1.19	0.778
Missing	1.01	0.86–1.18	0.916	1.09	0.89–1.33	0.390
Neutrophil: lymphocyte ratio	1.01	1.00–1.02	0.002	1.00	0.99–1.01	0.713
BMI						
18.5–25 kg/m^2^	Ref			Ref		
<18.5 kg/m^2^	2.08	1.48–2.93	<0.001	1.24	0.83–1.86	0.289
25–30 kg/m^2^	0.9	0.75–1.08	0.239	1.21	0.98–1.51	0.083
>30 kg/m^2^	0.65	0.54–0.79	<0.001	1.00	0.79–1.27	0.992
Missing	0.86	0.73–1.01	0.073	0.91	0.75–1.12	0.386
eGFR (mL/min/1.73 m^2^)						
>90	Ref			Ref		
60–89	0.87	0.72–1.05	0.136	0.73	0.58–0.91	0.005
45–59	0.99	0.80–1.21	0.898	0.71	0.55–0.92	0.008
30–44	1.31	1.05–1.63	0.018	0.71	0.54–0.94	0.016
15–29	1.75	1.34–2.28	<0.001	1.00	0.73–1.38	0.997
<15	1.84	1.34–2.52	<0.001	1.10	0.75–1.62	0.621
Missing	2.67	2.09–3.42	<0.001	1.64	1.07–2.53	0.024
Comorbidities						
Diabetes mellitus	1.16	1.02–1.33	0.025	0.92	0.78–1.09	0.336
Cardiovascular disease	1.29	1.15–1.46	<0.001	1.03	0.88–1.21	0.731
Respiratory disease	0.89	0.78–1.02	0.107	0.84	0.71–1.00	0.044
Cancer	1.23	1.00–1.50	0.045	1.11	0.88–1.40	0.361
Mental health	1.36	1.11–1.68	0.003	1.57	1.22–2.01	<0.001
Dementia	3.97	3.38–4.65	<0.001	1.73	1.39–2.16	<0.001

eGFR, Estimated Glomerular Filtration Rate by Modified Diet in Renal Disease formula.

## Discussion

### Interpretation of results

Age and frailty were independently associated with COVID-19 mortality. This is consistent with risk exhibited for nearly all other illnesses, and does not represent relative risk for COVID-19 compared with other illnesses; risk of dying increases with age and frailty (6) within ‘normal’ risk (24). However, increases in absolute mortality risk will be most pronounced in these groups, even if relative risk is equivalent to young or robust individuals. It is important to consider the results of likelihood testing, which demonstrated that age and frailty as individual predictors improved the model of fit. Risk continued to increase with increasing age and with increasing frailty. Therefore, the greatest risk will have been exhibited by the oldest and most frail patients. Underlying mechanisms for increased mortality with age and frailty with COVID-19 may include endothelial dysfunction leading to vasoconstriction and organ dysfunction (25, 26), heightened inflammation (27) and pro-coagulant state (25, 26, 28), dysregulated angiotensin-converting enzyme 2 activity promoting viral uptake (28–30) and immunesenescence (28). Immunesenescence is associated with immune system changes that are age-related (31, 32), frailty-related (33), or inactivity-related (34, 35).

Delirium was not independently predictive of mortality, but was associated with critical care admission; delirium itself is an illness severity marker. This may relate to exclusion of prevalent delirium cases, or represent different delirium pathophysiology with COVID-19 compared with other conditions. We demonstrated novel results that frailty, age, delirium, dementia and mental health diagnoses were independently associated with transitions of care in survivors i.e. adverse functional outcomes. Quality of life is individual and subjective, but increased dependency will have been hugely significant for many individuals. Transitions of care are also likely to have been associated with state-funded health and social care system costs, during a time of international economic recession, which has wider health impacts (36).

### What is the external validity of our results?

Previous studies assessing COVID-19 mortality with frailty showed mixed results. These have been predominantly small single site studies (9, 10, 37). Our results are consistent with another study including 1,410 UK-hospital and 154 Italian-hospital patients; sub-categorised CFS and age were independently associated with mortality (8). A second UK single site study involving 677 patients demonstrated increased mortality in CFS ≥ 6 (38). A small Italian study demonstrated that a Frailty Index was also predictive of mortality (37), even after removing co-morbidities from the index (39). However, a small UK study showed that age but not continuous CFS was predictive of mortality in univariable analysis; CFS was not included in multivariable analysis (40). Similar results were shown in another UK study (9). Differences may relate to under-powering in smaller studies, or how CFS was recorded or extracted from clinical records. In our study, few patients had missing CFS (11% versus 32% in the latter study) (9). However, these studies also assessed mortality following discharge, whereas we report mortality during index admission. Whilst this is important to distinguish, we do not consider this explanatory for differences; it is unlikely robust patients were more likely to have been discharged to die outside of hospital. As our data were censored at hospital discharge, this has also been accounted for.

Consistent with results elsewhere, male sex, inflammation and cancer were associated with mortality (18). However, BMI was not independently predictive of death, which is contrary to previous research (21), although high and low BMI were associated with critical care admission. Studies previously adjusted for age, sex and co-morbidities, but this is the first study to adjust for frailty. Both being underweight and obese have been associated with frailty (41).

Previous research demonstrated that delirium is a common COVID-19 presentation (12, 18). It is surprising that delirium did not predict mortality in our study. Delirium has been consistently associated with mortality in hospitalised patients with other illnesses (11, 42). A single-site Italian study demonstrated that delirium, diagnosed against reference criteria by geriatricians, was associated with 4-fold increased COVID-19 mortality risk (43). Under-recognition is unlikely to fully explain differences as prevalence was high in our study. Delirium was associated with critical care admission, which is consistent with a Brazilian study (44).

Frailty has been associated with prevalent delirium in COVID-19 (12) and other conditions (11). Incident delirium was not associated with frailty in this study. Additionally, dementia, a classical delirium risk factor, was not associated with risk. Higher risk was demonstrated with cardiovascular disease and illness severity. These differences in patient groups affected may explain differences in COVID-19 mortality with delirium compared with other conditions. Severely ill robust patients may have been as likely to develop delirium as frail patients, but less likely to die. We are not aware of other studies reporting transitions of care in patients with COVID-19. Studies outside of COVID-19 have shown that frailty and cognitive spectrum disorders are associated with increased risk of new discharge to a care home (45, 46).

### What is the internal validity of our results?

A major strength of our study is that it was large and multi-centre. This is the largest study to date evaluating how frailty and delirium relate to outcomes in hospitalised patients with COVID-19. Data collectors were not involved in analysis; statistical analysis was conducted by an independent statistician. We included large numbers of variables in multivariable analyses, which had been previously associated with adverse outcomes with COVID-19. We did not collect ethnicity data. Previous research identified that Black African or Caribbean and Bangladeshi individuals are at increased risk of adverse outcomes from COVID-19, although this also relates to socioeconomic status (47, 48). This personal information requires stricter safeguards (49), and is not internationally standardised.

Data collectors were provided delirium and frailty diagnosis guidance. Prospective data collection was encouraged and diagnoses were made by clinicians. Results provide predictive value of real-world delirium and frailty diagnoses. Given the high overall delirium prevalence, we consider it unlikely that under-diagnosis of delirium significantly impacted upon our overall results. Retrospectively identified data may be vulnerable to documentation errors, and we cannot rule out possibility of data entry errors via REDCap. To counteract this, data managers performed quality control checks on uploaded data, and contacted sites where data were missing or outlying values recorded.

Overall, missing data rates were low; discrete missing categories were included to account for those that were. The highest missing data rates related to BMI (height and weight). This could explain why obesity was not predictive of mortality; missing BMI was associated with mortality. It may have been most likely to be missing in most unwell or possibly most obese patients. Higher odds of mortality were demonstrated with missing CFS, which is consistent with previous studies (50). Multiple imputation has shown consistent effect of frailty on mortality in other populations (50).

Inclusion of hospitalised patients only is a limitation. The majority of people with COVID-19 had mild symptoms (51), particularly those who were young and robust; inclusion of community cases may have amplified association of frailty with mortality. Conversely, frail individuals may have died unexpectedly, or advance care planning decisions may have been made to avoid admission. Internationally, significant numbers of people who died from COVID-19 died in 24-h long-term care facilities (52, 53). We also recognise that our sample may not be internationally representative. As dissemination was via GeMRC (15, 16), more older adults may have been identified if clinicians were working on geriatric medicine wards. This in itself should not have affected main results; data collection was not biased towards outcomes.

### Recommendations for future research and clinical practice

Healthcare policy should recognise heightened vulnerability in older adults, particularly those living with frailty. Caution should be exhibited to ensure older adults with frailty are shielded from high risk COVID-19 exposure, such as ensuring isolation procedures during elective surgery admissions. Older and/or frail patients admitted with COVID-19 should undergo holistic assessment, ensuring treatment is proportionate and in accordance with their wishes. Research identifying underlying mechanisms of adverse outcomes with age and frailty may enable novel intervention development. It is vital older adults with frailty are adequately represented in all COVID-19 research. Vaccines and COVID-19 treatments may have different responsiveness with age or frailty.

Considering high odds of increased care in patients with frailty, urgent funding is needed to enhance community and hospital rehabilitation services (54). At present, longer-term consequences of COVID-19 are unknown. Chronic symptoms of fatigue and systemic upset have been reported even in community-dwelling previously robust adults (14). Further research should focus on understanding mechanisms and adverse recovery predictors, particularly in patients who developed acute sarcopenia (55, 56) or induced frailty (57).

## Conclusion

In this international multi-centre study, age, frailty and morbidity were independently associated with adverse outcomes with COVID-19. Patients who were older or more severely frail were more likely to die, less likely to be admitted to critical care, and more likely to require higher care levels on discharge in survivors. Increased awareness of importance of measuring frailty alongside age and co-morbidities in hospitalised patients will assist clinicians making holistic decisions involving treatment of reversible pathology, prevention of unwanted or burdensome treatment and early rehabilitation.

## Supplementary Material

aa-20-1502-File003_afab026

aa-20-1502-File004_afab026

## Notes

*Members of the Geriatric Medicine Research Collaborative

**Members of the Covid Collaborative

## Notes

* Members of the Geriatric Medicine Research Collaborative: Mustafa Alsahab Lucy Beishon Bryony Brown Elinor Burn Jenni K. Burton Natalie Cox Melanie Dani Muhammed Elhadi Sarah Freshwater Victoria Gaunt Adam Gordon Marie Goujon Matthew Hale Terry Hughes Thomas A. Jackson Benjamin Jelley Asma Khan Heena Khiroya Rajni Lal Katy Madden Laura Magill Jane Masoli Tahir Masud Lauren McCluskey Natalie McNeela Awolkhier Mohammedseid-Nurhussien Hannah Moorey Mary Ni Lochlainn Krishnarajah Nirantharakumar Kelvin Okoth Christopher N. Osuafor Katherine Patterson Grace ME Pearson Rita Perry Michala Pettitt Jennifer Pigott Thomas Pinkney Terence Quinn Abigail Reynolds Sarah Richardson Nik Sanyal Adam Seed Isobel Sleeman Chee Soo Claire Steves W. David Strain Joanne Taylor Kelli Torsney Carly Welch Daisy Wilson Miles Witham

^**^Members of the Covid Collaborative: Hossam Aldein S. Abd Elazeem Mohammed H. Abdelhafez Amir Abdelmalak Omar A. Abdelwahab Osama MAS Abdulhadi Olubayode Adewole Mohammed Ahmad Eltayeb A. Ahmed Hazem Ahmed Islam A. Ahmed Mertcan Akcay Yeşim Akdeniz Emrah Akın Carolyn Akladious Francesco Alessandri Ali Ali Abdulmalek Aljafari Abdulmoiz Aljafari Mohammed Al-Sadawi Lobna Al-Sodani Fatih Altintoprak Gitanjali Amaratungaz Jocelyn Amer Sylvia Amini Taha Amir Cheran Anandarajah Rachael Anders Muhammed H. Ansari Kingsley Appiah Jolene Atia Catherine Atkin Avinash Aujayeb Elsayed M. Awad Mohammed A. Azab Mohammad T. Azam Sally Aziz Ahmed Y. Azzam Laxmi Babar Laura Babb Manpreet Badh Clare Baguneid Emily Bailey Efstratia Baili Sarah Baldwin Ioannis Baloyiannis Moulinath Bannerjee Anna Barnard Fabio Barra Hannah Bashir Monica Bawor Zülfü Bayhan Lucy Beishon James Belcher Ravindra Belgamwar Corrina Bentley Amy Birchenough Yen Nee J. Bo Hayley R. Boden Ahmad Bouhuwaish Gioia Brachini Laura Bremner Hannah Bridgwater Catherine Bryant Gabrielle Budd Sharon Budd Adam Budzikoski Reem Bulla Antonio Buondonno Antonio Buondonno Nicole Burden Elinor Burn Hejab Butt Recayi Capoglu Andra Caracostea Rifa Cardoso Alexis Carr Milagros Carrasco-Prats Caterina Cattel Giancarlo Ceccarelli Giuseppe Cecere Alexandros Charalabopoulos Evelyn Charsley Hannah Cheney-Lowe Theodore Chevallier Asad J. Choudhry Flavia Ciccarone Pierfranco M. Cicerchia Bruno Cirillo Fatma D. Collins Victoria Comerford Ahmed Cordie Siobhan Coulter Nick Coulthard Catrin Cox Victoria Cox Andrew Crowe Jack Cullen Jean Cummings Niamh Cunningham Daniel Curley Hannah Currie Madeleine Daly Jay Darley Nikhita Dattani Spyridon Davakis Rowan Davies Gilda De Paola Giorgio De Toma Sergio Del Valle-Ruiz Benyamin Deldar Hakan Demir Arjun Desai Nirali Desai Alice Devaney Lindsey Dew Jugdeep Dhesi Maria Dias Gordon Dick Parveen Doddamani Gurinder Dogra Tina Doll Hannah C. Dooley Samiullah Dost Catherine Dotchin Hannah Dowell Ioan M. Drghita James M. Dundas Giulia Duranti Hiren Dusara Rajesh Dwivedi Adam H. Dyer Alison Eastaugh Elinor Edwards Shrouk M. Elghazaly Ahmed O. Elmehrath Hope Elrick Mostafa El-Shazly Alexander Emery Eric W. Etchill Sarah Evans Felicity Evison Cassandra Fairhead Margherita Faulkner Agnieszka Felska Antia Fernandez Pedro V. Fernández- Fernández Antonella Ferraiolo Simone Ferrero Enrico Fiori Necattin Firat Gracie Fisk Anna Fleck Giovanni B. Fonsi Alodia Gabre-Kidan Gaetano Gallo Ratnam Gandhi Madeleine Garner Nikolaos Georgiou Hannah Gerretsen Nourhan AA Ghannam Andrew Ghobrial Hedra Ghobrial Zaynub Ghufoor Jake Gibbon Georgia F. Gilbert Marie Giles Clara Giménez-Francés Emre Gonullu Amy Gray Joshua H. Gray Deirdre Green Charlotte Greene Ellanna Griffin Karla Griffith Anthony Grubb Yue Guan Daniel N. Guerero Ayushi Gupta Claudio Gustavino Laurenny Guzman Ahmed KM Hadreiez Jiannis Hajiioannou Deevia Hanji Deepthy Hari Madhavan Tarık Harmantepe Patrick Harrison Barbara Hart Aidan Haslam Victoria Haunton Elliott R. Haut Torben Heinsohn Lindsay Hennah Helal F. Hetta Alexander Hickman Abigail Hobill Patrick CP Hogan Vesna Hogan Elizabeth Holmes Katie Honney Katharine Hood Katherine Hopkinson Lara Howells Nicole Hrouda Danielle Hunsley William Hurst Rand A. Hussein Mohamed Eltaher AA Ibrahim Ishmam Ibtida Aina Ibukunoluwakitan Irem Ishlek Rishi Iyer Karl Jackson Rosie Jackson Ellen James Hayley Jarvis Sophie Jeffs Nathan Jenko Sasha Jeyakumar Shahriar Kabir Harjinder Kainth Jason Kalloo Akhil Kanzaria Amalia Karapanou Nuha Kardaman Sandeep Karthikeyan Anne Karunatilleke Mairead Kelly Nicola I. Kelly Hesham Khalid Haris Khan Muhammad S. Khan Matthew King Thomas Kneen Li Kok Chiara Kratochwila Aneliya Kuzeva Pierfrancesco Lapolla Rebecca Lau Kar Yee Law Aimee Leadbetter Gabriel Lee Helena Lee Helena Lee Gavriella Levinson Grace Lewis Theodore Liakakos Stephen Lim Danielle Lis Emma Livesey Pedro López-Morales Lily Lowes Eleanor Lunt Emily Lyon Suvira Madan Zeinab Majid Harsha Malapati Jade Man Baguiasri Mandane Sarah H. Manning Baris Mantoglu Nuria Martínez-Sanz William Marx Almontacer EB Masood Tom Maughan Jamie Mawhinney Dominic Maxfield Jordan Mayer Henry Maynard Claire McDonald Aine McGovern Sophie Mclachlan Esther Medina-Manuel Simona Meneghini Michelle Metcalf John Millwood-Hargrave Andrea Mingoli Kelvin Miu Fawsiya Mohamed Soha M. Mohamed Aliae AR Mohamed Hussein Abdulkader Mohammad Aaliya Mohammed Ahmed A. Momen Farhana Moomo Ismael Mora-Guzmán Lizzie Moriarty Hamilton Morrin Claire Morris Nicholas Moss Mohamed M. Moustafa Maria Mpoura Mohammed Mubin Ali Muhtaroglu Georgina Muir Stephanie Mulhern Daniel Muller Declan C. Murphy Bushra Muzammil Varun Nadkarni Mariam Albatoul Nageh Yasmin K. NasrEldin Wasim Nawaz Hanna Nguyen Cliona Ni Cheallaigh Alexander Noar Samuel North Favour Nwolu Alice O’Docherty Omoteniola Odutola Sinead O’Dwyer Olebu Ogochukwu Catherine O’Mahony Lia Orlando Marc Osterdahl Christina Page Ismini Panayotidis Shivam Pancholi Jessica Parkin Lauren C. Passby Patricia Pastor-Pérez Harnish Patel Shefali Patel Rose Penfold Rupini Perinpanathan Konstantinos Perivoliotis Teresa Perra Martha Pinkney Enrico Pinotti Alberto Porcu Angeline Price Francesco Pugliese Prabhleen Puri Sylvia Pytraczyk Yusra Qaiser Maria Qurashi Dina Radenkovic Thurkka Rajeswaran Sarah F. Rapaport Tahmina Razzak Lara Reilly Paul Reynolds Alexandra Richardson Amelia Roberts Amelia Roberts Charlotte Roberts-Rhodes Tanya Robinson Aldo Rocca Emily Ross-Skinner Miguel Ruiz-Marín Rebecca Ryall Alshaimaa M. Saad Mahmoud M. Saad Ambreen Sadiq Giuseppe Sammarco Michail A. Sampanis Hazel Sanghvi Paolo Sapienza Ross Sayers Luca Scott Michael Sen Mosab AA Shaban Kathleen T. Shakespeare Ellie Shaw Hannah Shaw Jonathan Sheldrake Sing Yang Sim Luigi Simonelli Nikolaos V. Sipsas Jarita Sivam Sri Sivarajan Jennifer Smith Fabio Speranza Claire Spice Amanda Stafford Katharine Stambollouian Kent A. Stevens Jack Stewart Emma Stratton Hannah Street Michael Surtees Emma Swinnerton Ahmed SA Taher Caroline Tait Amybel Taylor Miriam Thake Katie Thin Hannah Thould Thyn Thyn Benjaman To Hannah Tobiss Kathryn Toppley Liam Townsend Ellen Tullo George Tzovaras Anthony Umeadi Hrisheekesh Vaidya María Valero-Soriano Rosanna Varden Vittoria Vergani Dominique Vervoort Giuseppina Vescio Mark Vettasseri Madiha Virk Vaishali Vyas Joanne Wagland Stephanie Wallis Chloe Warner Eleanor Watkins Hannah Watson Rachael Webb Sarah H. Welsh Ruth West Elisha Whelan Julie Whitney Mark Whitsey Catherine Wilcock Iain Wilkinson David Williams Megan Williamson Ruth H. Willott Mettha Wimalasundera Yu Lelt Win Laura Winter Stephanie Worrall Rebecca Wright Natalie Yeo Eirene Yeung Merve Yigit Yasin A. Yildiz Humza Yusuf Martina Zambon Hein Zaw Omar Zein Elabedeen
