# Supplementary material for: Age and frailty are independently associated with increased COVID-19 mortality and increased care needs in survivors: results of an international multi-centre study
Source: Age Ageing. 2021 Feb 5;50(3):617–30. doi: 10.1093/ageing/afab026 (PMC7929433; doi:10.1093/ageing/afab026)
Supplement: aa-20-1502-File003_afab026 [file aa-20-1502-file003_afab026.pdf]

Table S1 – Data variables collected as part of this study

Variable	Categories/ units
Age	Years
Sex	Male<br>Female
Pregnancy (If female <60)	Yes<br>No
Clinical Frailty Scale	1. Fit<br>2. Well<br>3. Managing well<br>4. Vulnerable<br>5. Mildly frail<br>6. Moderately frail<br>7. Severely frail<br>8. Very severely frail<br>9. Terminally ill
Previous residence	Home no formal care<br>Home with formal care<br>24-hour long-term care facility
Known/suspected cases at residence	Yes<br>No<br>Unknown
Symptoms (any)	Fever<br>Cough/breathlessness<br>Confusion<br>Other
Prevalent delirium on admission	Yes<br>No
Comorbidities (any)	None<br>Diabetes Mellitus<br>Cardiovascular Disease<br>Respiratory disease<br>Cancer<br>Mental Health<br>Dementia<br>HIV<br>Other
Pre-admission prescription of any of:	ACE-inhibitors/ Angiotension-receptor blockers<br>Non-steroidal anti-inflammatory drugs (NSAIDs)<br>Steroids<br>Immunosuppressants<br>Chemotherapy<br>Antiretrovirals
If prescribed any of above, were these stopped on admission?	Yes<br>No
If not prescribed NSAIDs, steroids, or antiretrovirals, were these newly started?	Yes<br>No
Antibiotics commenced during admission?	None

	Oral<br>Intravenous
Number of minutes from admission to first antibiotic administration	Minutes
Intravenous fluids commenced on admission?	Yes<br>No
Total volume IV fluids in first 24 hours	Litres
Height	Metres
Weight	Kilograms
Heart rate	Beats/min
Blood pressure	mmHg
Oxygen saturations	%
Inspired oxygen concentration (FiO_2)	%
Temperature	^oC
Respiratory rate	Resps/min
Glasgow Coma Scale	Score (3 to 15)
Blood gas performed	Arterial<br>Venous
Blood gas results	pH<br>pO_2 (kPa)<br>pCO_2 (kPa)<br>FiO_2 (%)<br>HCO_3^- (mol/L)<br>Base excess<br>Lactate (mmol/L)
Serum urea concentration	mg/dL OR mmol/L
Serum creatinine concentration	mg/dL OR micromol/L
Haemoglobin	g/L
Lymphocyte count	10^9/L
Neutrophil count	10^9/L
C-reactive protein	mg/L
Alanine Aminotransferease	IU/L
Ferritin	ng/mL
D-Dimer	ng/mL
Troponin	ng/mL
Chest X-ray/ CT scan changes	None<br>Pneumonia<br>Acute Respiratory Distress Syndrome<br>Not performed
Confirmed diagnosis of COVID-19	Yes<br>No
Method used to confirm diagnosis	PCR<br>Antibody test
If negative, was there a strong clinical suspicion of COVID-19	Yes<br>No
Positive sputum detection for COVID-19	Yes<br>No<br>Not tested
Positive sputum detection for bacteria	Yes<br>No

	Not tested
Positive blood viraemia for COVID-19	Yes<br>No<br>Not tested
Positive blood culture for bacteria	Yes<br>No<br>Not tested
Was a Do Not Attempt Cardio-Pulmonary Resuscitation (DNACPR) decision made during admission?	Yes<br>No
Were there other treatment and escalation limitation decisions in place?	Yes<br>No
If limitations/DNACPR, were these in place before admission?	Yes<br>No
Incident delirium during admission	Yes<br>No
Death during admission	Yes<br>No
If death, days to death from admission	Days
If death, was end of life care administered?	Yes<br>No
If death, were family members present?	Yes<br>No
If death, preferred place of care?	Own home<br>Care home<br>Hospice<br>Hospital<br>Other
Critical care admission at any point?	Yes<br>No
Days admitted to critical care	Days
Emergency surgery during admission	Yes<br>No
If survived, days to discharge from admission	Days
Discharge destination	Own home with no formal care<br>Home with care<br>24-hour long-term care facility<br>Hospice<br>Rehabilitation/ step-down unit<br>Other
Was discharge delayed due to concerns about community spread of COVID-19?	Yes<br>No
By how many days was discharge delayed?	Days

Table S2 – Participating sites

Country	Site
Egypt	Alazher University Hospital
	Assiut University Hospital
	Minya General Hospital
	Minia University Hospital
Spain	Hospital General Reina Sofía
	Hospital Santa Bárbara
United Kingdom	Barnsley Hospital NHS Foundation Trust
	City Hospital, Birmingham
	Solihull Hospital
	Birmingham Heartlands Hospital
	Queen Elizabeth Hospital Birmingham
	Sandwell General Hospital
	University Hospitals Coventry and Warwickshire
	Royal Derby Hospital
	Royal Devon and Exeter Hospital
	Royal Bolton Hospital
	Queen Elizabeth Hospital Gateshead
	Glasgow Royal Infirmary
	Queen Elizabeth Hospital King's Lynn
	Leicester Royal Infirmary
	King's College Hospital
	Princess Royal University Hospital
	St Thomas' Hospital
	Tunbridge Wells Hospital
	Royal Victoria Infirmary
	Queens Hospital Romford
	Queens Medical Centre Nottingham
	Northumbria NHS Hospital Trust
	Queen Alexandra Hospital
	East Surrey Hospital
	Salford Royal Hospital
	Northern General Hospital
	South Tyneside District hospital
	Southampton General Hospital
	Harplands Hospital
	Sunderland Royal Hospital
	Good Hope Hospital
	Great Western Hospital
	Royal Wolverhampton Hospital
Greece	Laiko University Hospital
	General University Hospital of Larissa
Ireland	St. James's Hospital
Iraq	Zafaraniyah General Hospital
Italy	Antonio Cardarelli
	University 'Magna Graecia' of Catanzaro

	IRCCS Ospedale Policlinico San Martino
	Policlinico San Pietro
	Policlinico Umberto I
	Cliniche San Pietro, A.O.U. Sassari
Libya	Tobruk Medical Center
	Alkhadra Hospital
Saudi Arabia	King Abdullah Medical City Specialist Hospital
Sudan	Sharq Alneel Hospital
Turkey	Sakarya Faculty Of Medicine
United States of America	Johns Hopkins Hospital
	SUNY Downstate Brooklyn
	SUNY Upstate University Hospital

Figure S1 – Flow diagram of patient data excluded from analysis

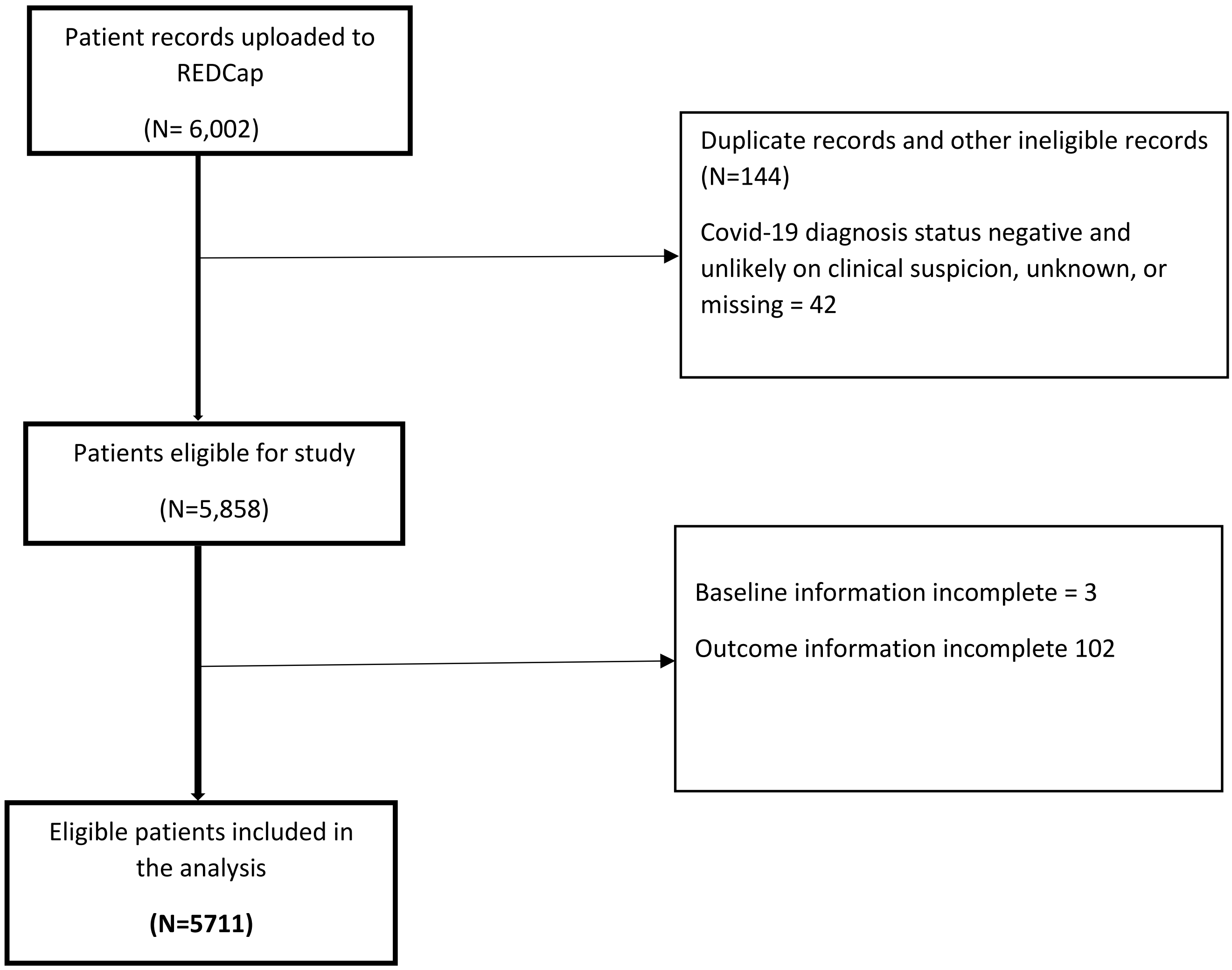

Table S3 – Cox regression with age and frailty as continuous variables

Characteristics	Model 1 HR	Model 1 95% CI	Model 1 P-value	Model 2 HR	Model 2 95% CI	Model 2 P-value	Model 3 HR	Model 3 95% CI	Model 3 P-value
**Delirium**									
No							Ref		
Yes							0.97	0.86 - 1.10	0.637
Missing							0.99	0.79 - 1.25	0.953
**Frailty (CFS score 1-8)**				1.17	1.12 - 1.21	<0.001	1.17	1.12 - 1.22	<0.001
**Age**	1.03	1.02 - 1.03	<0.001	1.02	1.01 - 1.03	<0.001	1.02	1.01 - 1.03	<0.001
**Sex**									
Female	Ref			Ref			Ref		
Male	1.22	1.09 - 1.37	0.001	1.25	1.11 - 1.42	<0.001	1.25	1.11 - 1.41	<0.001
**NEWS**									
0-4 (Low risk)	Ref			Ref			Ref		
5-6 (Medium risk)	1.47	1.26 - 1.71	<0.001	1.57	1.33 - 1.85	<0.001	1.57	1.33 - 1.85	<0.001
≥7 (High risk)	2.11	1.86 - 2.40	<0.001	2.13	1.85 - 2.45	<0.001	2.14	1.86 - 2.46	<0.001
Missing	1.87	1.58 - 2.22	<0.001	2.00	1.63 - 2.44	<0.001	2.00	1.63 - 2.44	<0.001
**CRP**									
<10	Ref			Ref			Ref		
10-40mg/L	1.24	0.92 - 1.66	0.152	1.23	0.89 - 1.68	0.204	1.23	0.89 - 1.68	0.206
>40mg/L	1.84	1.41 - 2.40	<0.001	1.83	1.37 - 2.43	<0.001	1.82	1.37 - 2.43	<0.001
Missing	2.11	1.47 - 3.02	<0.001	2.05	1.39 - 3.04	<0.001	2.05	1.38 - 3.04	<0.001
**Ferritin**									
< 100 ng/ml	Ref			Ref			Ref		
100 – 1000 ng/ml	1.77	1.11 - 2.83	0.017	1.91	1.16 - 3.15	0.011	1.91	1.16 - 3.15	0.011
>1000 ng/ml	1.65	1.01 - 2.69	0.045	1.90	1.13 - 3.19	0.015	1.90	1.13 - 3.19	0.016
Missing	1.92	1.21 - 3.03	0.005	2.02	1.24 - 3.28	0.005	2.02	1.24 - 3.28	0.005
**Alanine transferase**									
< 40 IU/L	Ref			Ref			Ref		
> 40 IU/L	1.11	0.97 - 1.27	0.139	1.11	0.96 - 1.29	0.171	1.11	0.96 - 1.29	0.164

Missing	0.92	0.79 - 1.07	0.282	0.94	0.80 - 1.11	0.489	0.94	0.80 - 1.11	0.501
**Neutrophil Lymphocyte Ratio**	1.00	1.00 - 1.01	0.011	1.00	1.00 - 1.01	0.022	1.00	1.00 - 1.01	0.021
**BMI**									
18.5-25 Kg/m^2	Ref			Ref			Ref		
<18.5kg/m^2	0.73	0.52 - 1.03	0.074	0.65	0.45 - 0.93	0.019	0.65	0.45 - 0.94	0.020
25-30Kg/m^2	0.90	0.75 - 1.07	0.225	0.93	0.77 - 1.12	0.458	0.93	0.77 - 1.12	0.452
>30Kg/m^2	0.98	0.82 - 1.18	0.852	1.00	0.82 - 1.22	1.000	1.00	0.82 - 1.22	0.999
Missing	1.46	1.27 - 1.67	<0.001	1.37	1.18 - 1.59	<0.001	1.37	1.18 - 1.60	<0.001
**eGFR (mL/min/1.73 m^2)**									
>90	Ref			Ref			Ref		
60-89	0.73	0.59 - 0.89	0.002	0.75	0.60 - 0.93	0.011	0.75	0.60 - 0.93	0.010
45-59	0.89	0.72 - 1.10	0.296	0.96	0.77 - 1.21	0.728	0.96	0.76 - 1.21	0.727
30-44	1.20	0.98 - 1.48	0.084	1.16	0.93 - 1.45	0.192	1.16	0.93 - 1.46	0.190
15-29	1.39	1.12 - 1.72	0.003	1.35	1.07 - 1.71	0.011	1.35	1.07 - 1.71	0.011
< 15	1.57	1.23 - 2.01	<0.001	1.44	1.10 - 1.88	0.009	1.44	1.10 - 1.88	0.009
Missing	0.96	0.65 - 1.44	0.858	0.93	0.60 - 1.43	0.733	0.93	0.61 - 1.43	0.742

Table S4 – Cox regression using models 1 and 2

	Model 1			Model 2		
	HR	95% CI	P-value	HR	95% CI	P-value
**Frailty Distribution**						
1-3				Ref		
4				1.62	1.30 - 2.00	<0.001
5				1.66	1.34 - 2.05	<0.001
6				1.74	1.42 - 2.13	<0.001
7				1.86	1.51 - 2.30	<0.001
8				2.96	2.24 - 3.92	<0.001
9				2.22	1.30 - 3.80	0.004
Missing				2.32	1.88 - 2.85	<0.001
**Age Distribution**						
18- 49 years	Ref			Ref		
50-64 years	1.99	1.41 - 2.82	<0.001	1.93	1.36 - 2.73	<0.001
65-80 years	3.57	2.58 - 4.95	<0.001	2.90	2.08 - 4.04	<0.001
> 80 years	4.61	3.31 - 6.43	<0.001	3.52	2.50 - 4.95	<0.001
**Sex**						
Female	Ref			Ref		
Male	1.20	1.07 - 1.34	0.002	1.22	1.09 - 1.37	<0.001
**NEWS**						
0-4 (Low risk)	Ref			Ref		
5-6 (Medium risk)	1.46	1.25 - 1.70	<0.001	1.53	1.31 - 1.78	<0.001
≥7 (High risk)	2.10	1.84 - 2.38	<0.001	2.11	1.86 - 2.41	<0.001
Missing	1.86	1.57 - 2.21	<0.001	1.71	1.43 - 2.03	<0.001
**CRP**						
< 10mg/l	Ref			Ref		
10-40mg/l	1.24	0.92 - 1.65	0.154	1.23	0.92 - 1.64	0.172
>40mg/l	1.84	1.41 - 2.40	<0.001	1.85	1.42 - 2.41	<0.001
Missing	2.10	1.47 - 3.01	<0.001	2.22	1.55 - 3.17	<0.001

**Ferritin**						
< 100 ng/ml	Ref			Ref		
100 – 1000 ng/ml	1.70	1.06 - 2.72	0.027	1.82	1.13 - 2.92	0.013
>1000 ng/ml	1.59	0.97 - 2.58	0.064	1.73	1.06 - 2.82	0.028
Missing	1.83	1.16 - 2.89	0.010	1.88	1.19 - 2.97	0.007
**Alanine transferase**						
< 40 IU/L	Ref			Ref		
> 40 IU/L	1.10	0.96 - 1.26	0.156	1.16	1.01 - 1.33	0.032
Missing	0.93	0.80 - 1.09	0.371	0.96	0.82 - 1.12	0.586
**Neutrophil: Lymphocyte Ratio**	1.00	1.00 - 1.01	0.006	1.00	1.00 - 1.01	0.015
**BMI**						
18.5-25 kg/m^2	Ref			Ref		
<18.5kg/m^2	0.77	0.55 - 1.08	0.129	0.73	0.52 - 1.03	0.078
25-30kg/m^2	0.88	0.74 - 1.05	0.170	0.94	0.79 - 1.13	0.533
>30kg/m^2	0.99	0.82 - 1.19	0.898	1.02	0.85 - 1.23	0.798
Missing	1.48	1.29 - 1.70	<0.001	1.46	1.27 - 1.68	<0.001
**eGFR (mL/min/1.73 m^2)**						
>90	Ref			Ref		
60-89	0.74	0.60 - 0.91	0.004	0.75	0.61 - 0.92	0.006
45-59	0.92	0.74 - 1.13	0.428	0.94	0.76 - 1.16	0.556
30-44	1.23	1.00 - 1.51	0.049	1.24	1.01 - 1.52	0.041
15-29	1.43	1.15 - 1.77	0.001	1.38	1.12 - 1.71	0.003
< 15	1.60	1.25 - 2.03	<0.001	1.51	1.18 - 1.92	0.001
Missing	1.00	0.67 - 1.48	0.982	0.98	0.66 - 1.47	0.938
**Comorbidities**						
Diabetes Mellitus	1.07	0.96 - 1.20	0.225	1.07	0.95 - 1.20	0.248
Cardiovascular Disease	1.10	0.98 - 1.23	0.112	1.09	0.98 - 1.23	0.122
Respiratory Disease	0.97	0.87 - 1.10	0.660	0.95	0.84 - 1.07	0.389
Cancer	1.22	1.05 - 1.41	0.007	1.21	1.04 - 1.39	0.011

Mental Health	0.94	0.77 - 1.14	0.503	0.86	0.71 - 1.05	0.138
Dementia	1.20	1.05 - 1.36	0.006	1.06	0.93 - 1.22	0.382

Table S5 – Cox regression analysis including age and frailty interactions

	HR	95% CI	P-value
**Delirium**			
No	Ref		
Yes	0.98	0.87 – 1.10	0.690
Missing	0.83	0.67 – 1.02	0.073
**Frailty*Age Interaction**			
CFS 1-3, 18-49 years	Ref		
CFS 1-3, 50-64 years	2.63	1.54 – 4.49	<0.001
CFS 1-3, 65-80 years	4.68	2.76 – 7.91	<0.001
CFS 1-3, > 80 years	6.46	3.66 – 11.42	<0.001
CFS 4-6, 18-49 years	4.06	1.99 – 8.27	<0.001
CFS 4-6, 50-64 years	6.77	3.85 – 11.88	<0.001
CFS 4-6 65-80 years	6.85	4.12 – 11.37	<0.001
CFS 4-6 > 80 years	8.78	5.30 – 14.54	<0.001
CFS 7-8, 18-49 years	6.78	2.49 – 18.49	<0.001
CFS 7-8, 50-64 years	6.28	2.86 – 13.78	<0.001
CFS 7-8, 65-80 years	9.68	5.70 – 16.42	<0.001
CFS 7-8, > 80 years	10.15	6.07 – 16.98	<0.001
Missing CFS, 18-49 years	3.77	1.47 – 9.65	0.006
Missing CFS, 50-64 years	4.99	2.58 – 9.65	<0.001
Missing CFS, 65-80 years	9.94	5.78 – 17.10	<0.001
Missing CFS, 1-3 > 80 years	13.62	8.02 – 23.15	<0.001
**Sex**			
Female	Ref		
Male	1.21	1.08 – 1.36	0.001
**NEWS**			
0-4 (Low risk)	Ref		
5-6 (Medium risk)	1.52	1.31 – 1.78	<0.001
≥7 (High risk)	2.11	1.85 – 2.41	<0.001
Missing	1.78	1.49 – 2.13	<0.001
**CRP**			
< 10mg/l	Ref		
10-40mg/l	1.23	0.92 – 1.64	0.170
>40mg/l	1.89	1.45 – 2.47	<0.001
Missing	2.19	1.53 – 3.14	<0.001
**Ferritin**			
< 100 ng/ml	Ref		
100 – 1000 ng/ml	1.73	1.08 – 2.77	0.023
>1000 ng/ml	1.71	1.05 – 2.79	0.031
Missing	2.19	1.53 – 3.14	<0.001
**Alanine transferase**			

< 40 IU/L	Ref		
> 40 IU/L	1.14	0.99 – 1.30	0.063
Missing	0.95	0.81 – 1.10	0.483
**Neutrophil: Lymphocyte Ratio**	1.00	1.00 – 1.00	0.027
**BMI**			
18.5-25 kg/m^2	Ref		
<18.5kg/m^2	0.71	0.50 – 1.00	0.052
25-30kg/m^2	0.93	0.77 – 1.11	0.393
>30kg/m^2	1.01	0.84 – 1.22	0.883
Missing	1.40	1.21 – 1.61	<0.001
**eGFR (mL/min/1.73 m^2)**			
>90	Ref		
60-89	0.74	0.60 – 0.91	0.004
45-59	0.93	0.75 – 1.15	0.518
30-44	1.22	0.99 – 1.51	0.057
15-29	1.37	1.10 – 1.70	0.004
< 15	1.50	1.17 – 1.91	0.001
Missing	0.98	0.66 – 1.46	0.927
**Comorbidities**			
Diabetes Mellitus	1.07	0.96 – 1.20	0.217
Cardiovascular Disease	1.09	0.97 – 1.23	0.137
Respiratory Disease	0.94	0.83 – 1.05	0.276
Cancer	1.19	1.02 – 1.37	0.022
Mental Health	0.86	0.70 – 1.04	0.122
Dementia	1.10	0.96 – 1.26	0.177

Table S6 – Sensitivity analysis including only aged ≥65 years-old

	Model 1			Model 2			Model 3		
	HR	95% CI	P-value	HR	95% CI	P-value	HR	95% CI	P-value
**Delirium**									
No							Ref		
Yes							0.99	0.88 - 1.12	0.866
Missing							0.74	0.58 - 0.93	0.011
**Frailty Distribution**									
1-3				Ref			Ref		
4				1.44	1.13 - 1.83	0.003	1.44	1.13 - 1.83	0.003
5				1.40	1.11 - 1.78	0.005	1.42	1.12 - 1.80	0.004
6				1.53	1.22 - 1.92	<0.001	1.55	1.24 - 1.94	<0.001
7				1.61	1.28 - 2.03	<0.001	1.63	1.29 - 2.06	<0.001
8				2.65	1.97 - 3.56	<0.001	2.69	2.00 - 3.62	<0.001
9				1.95	1.11 - 3.43	0.020	2.10	1.19 - 3.69	0.010
Missing				2.14	1.69 - 2.71	<0.001	2.24	1.77 - 2.85	<0.001
Age (> 65 years)	1.02	1.01 - 1.02	<0.001	1.01	1.00 - 1.02	0.002	1.01	1.00 - 1.02	0.002
**Sex**									
Female	Ref			Ref			Ref		
Male	1.25	1.11 - 1.41	<0.001	1.27	1.12 - 1.43	<0.001	1.27	1.12 - 1.43	<0.001
**NEWS**									
0-4 (Low risk)	Ref			Ref			Ref		
5-6 (Medium risk)	1.51	1.29 - 1.78	<0.001	1.57	1.34 - 1.85	<0.001	1.58	1.34 - 1.85	<0.001
≥7 (High risk)	2.08	1.81 - 2.39	<0.001	2.09	1.82 - 2.40	<0.001	2.08	1.81 - 2.39	<0.001
Missing	1.84	1.53 - 2.21	<0.001	1.69	1.40 - 2.04	<0.001	1.74	1.44 - 2.10	<0.001
**CRP**									
< 10mg/l	Ref			Ref			Ref		
10-40mg/l	1.16	0.86 - 1.58	0.324	1.15	0.85 - 1.56	0.372	1.16	0.85 - 1.57	0.345
>40mg/l	1.70	1.29 - 2.24	<0.001	1.70	1.29 - 2.24	<0.001	1.72	1.31 - 2.27	<0.001

Missing	1.90	1.29 - 2.82	0.001	1.99	1.34 - 2.94	0.001	2.00	1.35 - 2.96	0.001
**Ferritin**									
< 100 ng/ml	Ref			Ref			Ref		
100 – 1000 ng/ml	1.67	1.03 - 2.72	0.038	1.75	1.07 - 2.84	0.025	1.75	1.07 - 2.84	0.025
>1000 ng/ml	1.63	0.98 - 2.72	0.059	1.72	1.03 - 2.86	0.038	1.74	1.05 - 2.90	0.033
Missing	1.79	1.12 - 2.86	0.015	1.81	1.13 - 2.90	0.014	1.84	1.15 - 2.94	0.011
**Alanine transferase**									
< 40 IU/L	Ref			Ref			Ref		
> 40 IU/L	1.17	1.01 - 1.35	0.040	1.22	1.05 - 1.41	0.010	1.22	1.05 - 1.41	0.009
Missing	0.93	0.79 - 1.09	0.349	0.96	0.81 - 1.12	0.577	0.94	0.80 - 1.11	0.465
**Neutrophil: Lymphocyte Ratio**	1.01	1.00 - 1.01	0.004	1.00	1.00 - 1.01	0.017	1.00	1.00 - 1.01	0.021
**BMI**									
18.5-25 Kg/m^2	Ref			Ref			Ref		
<18.5kg/m^2	0.73	0.51 - 1.04	0.083	0.71	0.49 - 1.02	0.062	0.70	0.48 - 1.00	0.051
25-30Kg/m^2	0.90	0.74 - 1.09	0.287	0.95	0.79 - 1.15	0.622	0.95	0.79 - 1.15	0.618
>30Kg/m^2	1.05	0.85 - 1.29	0.665	1.07	0.87 - 1.31	0.545	1.08	0.87 - 1.33	0.493
Missing	1.51	1.30 - 1.74	<0.001	1.50	1.29 - 1.73	<0.001	1.45	1.25 - 1.68	<0.001
**eGFR (mL/min/1.73 m^2)**									
**>90**	Ref			Ref			Ref		
60-89	0.77	0.61 - 0.97	0.027	0.78	0.62 - 0.99	0.039	0.78	0.62 - 0.99	0.038
45-59	0.93	0.73 - 1.19	0.573	0.95	0.75 - 1.20	0.659	0.94	0.74 - 1.19	0.598
30-44	1.19	0.94 - 1.50	0.149	1.20	0.95 - 1.52	0.120	1.18	0.94 - 1.50	0.156
15-29	1.38	1.08 - 1.76	0.009	1.35	1.06 - 1.72	0.015	1.33	1.05 - 1.70	0.020
< 15	1.54	1.17 - 2.02	0.002	1.46	1.11 - 1.92	0.007	1.47	1.12 - 1.93	0.006
Missing	0.98	0.62 - 1.54	0.922	0.98	0.62 - 1.54	0.920	1.01	0.64 - 1.59	0.962
**Comorbidities**									
Diabetes Mellitus	1.01	0.90 - 1.14	0.855	1.01	0.89 - 1.14	0.889	1.01	0.89 - 1.14	0.901
Cardiovascular Disease	1.05	0.93 - 1.18	0.475	1.05	0.93 - 1.19	0.443	1.03	0.91 - 1.16	0.670
Respiratory Disease	0.94	0.83 - 1.07	0.377	0.92	0.81 - 1.05	0.208	0.91	0.80 - 1.04	0.159

Cancer	1.22	1.05 - 1.41	0.011	1.21	1.04 - 1.41	0.013	1.21	1.04 - 1.40	0.016
Mental Health	0.89	0.72 - 1.10	0.283	0.83	0.67 - 1.03	0.090	0.82	0.66 - 1.02	0.071
Dementia	1.19	1.05 - 1.36	0.008	1.08	0.94 - 1.24	0.287	1.07	0.93 - 1.23	0.345

Table S7 – Sensitivity analysis including only UK data

	Model 1			Model 2			Model 3		
	HR	95% CI	P value	HR	95% CI	P value	HR	95% CI	P value
**Delirium**									
No							Ref		
Yes							0.97	0.86 - 1.09	0.603
Missing							0.80	0.65 - 0.99	0.039
**Frailty Distribution**									
1-3				Ref			Ref		
4				1.46	1.16 - 1.85	0.001	1.48	1.17 - 1.86	0.001
5				1.54	1.23 - 1.93	<0.001	1.57	1.25 - 1.96	<0.001
6				1.62	1.31 - 2.00	<0.001	1.65	1.33 - 2.04	<0.001
7				1.73	1.39 - 2.15	<0.001	1.76	1.41 - 2.19	<0.001
8				2.77	2.08 - 3.69	<0.001	2.83	2.12 - 3.77	<0.001
9				2.11	1.23 - 3.63	0.007	2.24	1.30 - 3.86	0.004
Missing				2.18	1.76 - 2.71	<0.001	2.27	1.83 - 2.82	<0.001
**Age Distribution**									
18- 49 years	Ref			Ref			Ref		
50-64 years	1.99	1.35 - 2.96	0.001	1.91	1.29 - 2.83	0.001	1.93	1.30 - 2.86	0.001
65-80 years	3.82	2.64 - 5.53	<0.001	3.16	2.17 - 4.60	<0.001	3.19	2.19 - 4.64	<0.001
> 80 years	4.91	3.38 - 7.15	<0.001	3.86	2.63 - 5.67	<0.001	3.91	2.66 - 5.74	<0.001
**Sex**									
Female	Ref			Ref			Ref		
Male	1.20	1.07 - 1.34	0.002	1.21	1.08 - 1.36	0.001	1.21	1.08 - 1.36	0.001
**New score**									
0-4 (Low risk)	Ref			Ref			Ref		
5-6 (Medium risk)	1.40	1.20 - 1.64	<0.001	1.47	1.25 - 1.73	<0.001	1.47	1.25 - 1.73	<0.001
≥7 (High risk)	2.01	1.75 - 2.30	<0.001	2.03	1.77 - 2.33	<0.001	2.03	1.77 - 2.32	<0.001
Missing	1.82	1.54 - 2.17	<0.001	1.68	1.41 - 2.01	<0.001	1.72	1.44 - 2.06	<0.001

**CRP**									
< 10mg/l	Ref			Ref			Ref		
10-40mg/l	1.16	0.86 - 1.56	0.334	1.15	0.86 - 1.56	0.346	1.16	0.86 - 1.57	0.318
>40mg/l	1.74	1.32 - 2.27	<0.001	1.76	1.34 - 2.30	<0.001	1.78	1.36 - 2.33	<0.001
Missing	1.96	1.33 - 2.89	0.001	2.06	1.39 - 3.03	<0.001	2.07	1.40 - 3.05	<0.001
**Ferritin**									
< 100 ng/ml	Ref			Ref			Ref		
100 – 1000 ng/ml	1.64	1.02 - 2.63	0.040	1.75	1.09 - 2.81	0.021	1.75	1.09 - 2.81	0.021
>1000 ng/ml	1.54	0.94 - 2.52	0.083	1.68	1.02 - 2.75	0.040	1.69	1.03 - 2.77	0.037
Missing	1.68	1.06 - 2.65	0.027	1.72	1.09 - 2.72	0.021	1.73	1.10 - 2.75	0.019
**Alanine transferase**									
< 40 IU/L	Ref			Ref			Ref		
> 40 IU/L	1.11	0.97 - 1.28	0.129	1.17	1.02 - 1.35	0.026	1.17	1.02 - 1.35	0.026
Missing	0.91	0.78 - 1.06	0.232	0.93	0.80 - 1.09	0.385	0.92	0.79 - 1.08	0.326
**Neutrophil: lymphocyte ratio**	1.01	1.00 - 1.01	0.001	1.00	1.00 - 1.01	0.007	1.00	1.00 - 1.01	0.009
**BMI**									
18.5-25 Kg/m^2	Ref			Ref			Ref		
<18.5kg/m^2	0.73	0.51 - 1.03	0.076	0.70	0.50 - 1.00	0.052	0.70	0.49 - 0.99	0.044
25-30Kg/m^2	0.91	0.75 - 1.10	0.329	0.96	0.80 - 1.17	0.704	0.97	0.80 - 1.17	0.720
>30Kg/m^2	0.98	0.81 - 1.19	0.844	1.01	0.83 - 1.23	0.904	1.02	0.84 - 1.24	0.875
Missing	1.48	1.28 - 1.71	<0.001	1.46	1.27 - 1.69	<0.001	1.43	1.23 - 1.65	<0.001
**eGFR (mL/min/1.73 m^2)**									
**>90**	Ref			Ref			Ref		
60-89	0.77	0.62 - 0.96	0.02	0.78	0.63 - 0.98	0.030	0.78	0.63 - 0.97	0.027
45-59	0.95	0.76 - 1.19	0.665	0.97	0.78 - 1.22	0.815	0.96	0.77 - 1.21	0.735
30-44	1.28	1.03 - 1.60	0.026	1.30	1.05 - 1.63	0.018	1.29	1.03 - 1.61	0.026
15-29	1.48	1.18 - 1.86	0.001	1.45	1.15 - 1.82	0.002	1.44	1.14 - 1.81	0.002
< 15	1.62	1.25 - 2.09	<0.001	1.55	1.20 - 2.00	0.001	1.55	1.20 - 2.00	0.001
Missing	1.09	0.69 - 1.72	0.701	1.06	0.67 - 1.67	0.805	1.10	0.70 - 1.73	0.691
**Comorbidities**									

Diabetes Mellitus	1.08	0.96 - 1.22	0.188	1.09	0.97 - 1.22	0.152	1.09	0.97 - 1.23	0.144
Cardiovascular Disease	1.09	0.97 - 1.22	0.167	1.09	0.96 - 1.22	0.173	1.07	0.95 - 1.21	0.264
Respiratory Disease	0.98	0.86 - 1.10	0.688	0.96	0.85 - 1.08	0.487	0.95	0.84 - 1.07	0.400
Cancer	1.27	1.09 - 1.47	0.002	1.26	1.09 - 1.47	0.002	1.25	1.08 - 1.46	0.003
Mental Health	0.99	0.81 - 1.20	0.909	0.92	0.75 - 1.12	0.392	0.91	0.75 - 1.11	0.348
Dementia	1.21	1.06 - 1.38	0.005	1.08	0.94 - 1.24	0.271	1.08	0.94 - 1.24	0.290

Table S8 – Logistic regression for critical care admission using models 1 and 2

	Model 1			Model 2		
	HR	95% CI	P-value	HR	95% CI	P-value
**Frailty Distribution**						
1-3				Ref		
4				0.76	0.55 - 1.05	0.096
5				0.44	0.29 - 0.66	<0.001
6				0.36	0.23 - 0.57	<0.001
7				0.13	0.07 - 0.27	<0.001
8				0.07	0.01 - 0.50	0.009
9				-	-	-
Missing				0.56	0.39 - 0.81	0.002
**Age Distribution**						
18- 49 years	Ref			Ref		
50-64 years	0.99	0.76 - 1.29	0.947	1.05	0.80 - 1.37	0.740
65-80 years	0.55	0.41 - 0.74	<0.001	0.75	0.55 - 1.01	0.057
> 80 years	0.09	0.06 - 0.15	<0.001	0.16	0.10 - 0.25	<0.001
**Sex**						
Female	Ref			Ref		
Male	1.27	1.03 - 1.58	0.028	1.21	0.97 - 1.51	0.083
**NEWS**						
0-4 (Low risk)	Ref			Ref		
5-6 (Medium risk)	2.06	1.57 - 2.70	<0.001	2.00	1.52 - 2.63	<0.001
≥7 (High risk)	3.92	3.08 - 4.99	<0.001	4.23	3.31 - 5.41	<0.001
Missing	3.25	2.29 - 4.62	<0.001	3.57	2.48 - 5.13	<0.001
**CRP**						
< 10mg/l	Ref			Ref		
10-40mg/l	1.46	0.88 - 2.41	0.138	1.46	0.89 - 2.42	0.137
>40mg/l	1.86	1.18 - 2.93	0.008	1.83	1.16 - 2.90	0.010
Missing	4.07	2.23 - 7.44	<0.001	3.71	2.02 - 6.84	<0.001
**Ferritin**						
< 100 ng/ml	Ref			Ref		
100 – 1000 ng/ml	2.45	1.17 - 5.14	0.018	2.18	1.03 - 4.61	0.042
>1000 ng/ml	4.54	2.13 - 9.66	<0.001	3.98	1.85 - 8.55	<0.001
Missing	0.98	0.47 - 2.04	0.963	0.92	0.44 - 1.93	0.831
**Alanine transferase**						
< 40 IU/L	Ref			Ref		
> 40 IU/L	1.24	1.00 - 1.54	0.055	1.19	0.96 - 1.49	0.116
Missing	0.73	0.53 - 1.02	0.064	0.69	0.50 - 0.97	0.031
**Neutrophil: Lymphocyte Ratio**	1.01	1.00 - 1.01	0.191	1.01	1.00 - 1.02	0.064
**BMI**						

18.5-25 kg/m^2	Ref			Ref		
<18.5kg/m^2	0.20	0.05 - 0.87	0.031	0.23	0.05 - 0.98	0.047
25-30kg/m^2	1.47	1.09 - 1.99	0.011	1.37	1.01 - 1.86	0.045
>30kg/m^2	1.58	1.18 - 2.12	0.002	1.50	1.12 - 2.03	0.007
Missing	0.80	0.59 - 1.08	0.152	0.83	0.61 - 1.12	0.224
**eGFR (mL/min/1.73 m^2)**						
>90	Ref			Ref		
60-89	0.91	0.68 - 1.21	0.516	0.88	0.66 - 1.18	0.398
45-59	1.29	0.93 - 1.79	0.132	1.25	0.90 - 1.75	0.187
30-44	1.24	0.86 - 1.78	0.254	1.23	0.85 - 1.78	0.279
15-29	1.34	0.88 - 2.03	0.173	1.39	0.90 - 2.12	0.135
< 15	0.94	0.58 - 1.52	0.796	1.04	0.63 - 1.71	0.870
Missing	0.94	0.52 - 1.70	0.833	0.97	0.53 - 1.78	0.931
**Comorbidities**						
Diabetes Mellitus	1.13	0.91 - 1.40	0.283	1.16	0.93 - 1.45	0.179
Cardiovascular Disease	1.17	0.94 - 1.45	0.153	1.21	0.97 - 1.51	0.089
Respiratory Disease	0.98	0.78 - 1.23	0.860	1.03	0.82 - 1.30	0.790
Cancer	1.05	0.76 - 1.46	0.763	1.15	0.82 - 1.62	0.409
Mental Health	0.86	0.60 - 1.23	0.400	1.00	0.69 - 1.45	0.995
Dementia	0.17	0.08 - 0.35	<0.001	0.33	0.16 - 0.71	0.005

Table S9 – Odds ratios derived from logistic regression for secondary outcomes of incident delirium

	Univariable			Multivariable		
	**OR**	**95% CI**	**P-value**	**OR**	**95% CI**	**P-value**
**Frailty Distribution**						
1-3	Ref			Ref		
4	1.49	1.04 - 2.12	0.028	1.07	0.72 - 1.60	0.745
5	1.43	1.01 - 2.04	0.045	1.06	0.71 - 1.58	0.779
6	1.86	1.39 - 2.49	<0.001	1.24	0.86 - 1.79	0.258
7	1.97	1.46 - 2.66	<0.001	1.38	0.94 - 2.03	0.102
8	2.48	1.51 - 4.06	<0.001	1.35	0.75 - 2.44	0.317
9	1.17	0.28 - 4.97	0.830	0.41	0.05 - 3.10	0.386
Missing	1.21	0.84 - 1.75	0.299	0.96	0.63 - 1.45	0.831
**Age Distribution**						
18- 49 years	Ref			Ref		
50-64 years	1.57	1.01 - 2.42	0.045	1.29	0.82 - 2.03	0.272
65-80 years	2.07	1.38 - 3.09	<0.001	1.59	1.01 - 2.51	0.045
> 80 years	2.93	1.99 - 4.30	<0.001	2.21	1.37 - 3.59	0.001
**Sex**						
Female	Ref			Ref		
Male	1.24	1.01 - 1.51	0.037	1.26	1.01 - 1.57	0.039
**NEWS**						
0-4 (Low risk)	Ref			Ref		
5-6 (Medium risk)	1.08	0.81 - 1.43	0.600	1.02	0.76 - 1.38	0.876
≥7 (High risk)	1.76	1.40 - 2.21	<0.001	1.52	1.18 - 1.96	0.001
Missing	1.40	1.00 - 1.97	0.050	1.32	0.91 - 1.91	0.141
**CRP**						
< 10mg/l	Ref			Ref		
10-40mg/l	1.40	0.93 - 2.13	0.110	1.21	0.78 - 1.89	0.392
>40mg/l	1.49	1.03 - 2.17	0.034	1.15	0.77 - 1.72	0.505
Missing	0.82	0.46 - 1.45	0.499	0.96	0.47 - 1.98	0.923
**Ferritin**						
< 100 ng/ml	Ref			Ref		
100 – 1000 ng/ml	1.40	0.93 - 2.13	0.110	1.46	0.73 - 2.92	0.283
>1000 ng/ml	1.49	1.03 - 2.17	0.034	1.62	0.78 - 3.36	0.197
Missing	0.82	0.46 - 1.45	0.499	1.02	0.52 - 2.00	0.945
**Alanine transferase**						
< 40 IU/L	Ref			Ref		
> 40 IU/L	1.08	0.85 - 1.37	0.552	1.17	0.90 - 1.52	0.248
Missing	0.79	0.60 - 1.04	0.088	0.98	0.72 - 1.32	0.873
**Neutrophil: Lymphocyte Ratio**	1.01	1.00 - 1.02	0.002	1.01	1.00 - 1.01	0.175
**BMI**						

18.5-25 kg/m^2	Ref			Ref		
<18.5kg/m^2	1.46	0.85 - 2.53	0.172	1.25	0.70 - 2.22	0.455
25-30kg/m^2	0.91	0.66 - 1.25	0.570	0.94	0.67 - 1.31	0.695
>30kg/m^2	0.95	0.70 - 1.31	0.767	1.15	0.82 - 1.62	0.423
Missing	1.16	0.89 - 1.51	0.260	1.06	0.80 - 1.40	0.688
**eGFR (mL/min/1.73 m^2)**						
>90	Ref			Ref		
60-89	0.84	0.61 - 1.16	0.291	0.75	0.53 - 1.05	0.089
45-59	0.94	0.66 - 1.33	0.706	0.71	0.49 - 1.03	0.074
30-44	1.39	0.99 - 1.94	0.055	0.90	0.62 - 1.30	0.575
15-29	1.50	1.04 - 2.17	0.031	0.88	0.58 - 1.32	0.535
< 15	1.14	0.72 - 1.80	0.577	0.76	0.46 - 1.27	0.299
Missing	0.59	0.35 - 0.99	0.046	0.58	0.25 - 1.35	0.210
**Comorbidities**						
Diabetes Mellitus	1.20	0.97 - 1.48	0.087	1.04	0.82 - 1.30	0.763
Cardiovascular Disease	1.67	1.37 - 2.04	<0.001	1.29	1.03 - 1.62	0.028
Respiratory Disease	1.67	1.37 - 2.04	<0.001	1.13	0.90 - 1.42	0.278
Cancer	1.00	0.74 - 1.37	0.976	0.89	0.64 - 1.24	0.492
Mental Health	1.00	0.70 - 1.42	0.993	1.07	0.75 - 1.55	0.701
Dementia	1.44	1.13 - 1.84	0.003	1.12	0.83 - 1.49	0.460

NEWS = National Early Warning Score; CRP = C-Reactive Protein; BMI = Body Mass Index; eGFR = Estimated Glomerular Filtration Rate by Modified Diet in Renal Disease formula

Table S10 – Logistic regression for incident delirium using model 1

	HR	95% CI	P-value
**Age Distribution**			
18- 49 years	Ref		
50-64 years	1.30	0.83 - 2.05	0.255
65-80 years	1.70	1.10 - 2.63	0.017
> 80 years	2.48	1.58 - 3.89	<0.001
**Sex**			
Female	Ref		
Male	1.24	1.00 - 1.55	0.051
**NEWS**			
0-4 (Low risk)	Ref		
5-6 (Medium risk)	1.03	0.77 - 1.39	0.838
≥7 (High risk)	1.55	1.21 - 2.00	0.001
Missing	1.29	0.90 - 1.85	0.171
**CRP**			
< 10mg/l	Ref		
10-40mg/l	1.23	0.79 - 1.91	0.472
>40mg/l	1.16	0.77 - 1.74	0.909
Missing	0.96	0.47 - 1.97	0.337
**Ferritin**			
< 100 ng/ml	Ref		
100 – 1000 ng/ml	1.40	0.70 - 2.79	0.337
>1000 ng/ml	1.56	0.75 - 3.23	0.231
Missing	1.00	0.51 - 1.94	0.995
**Alanine transferase**			
< 40 IU/L	Ref		
> 40 IU/L	1.15	0.89 - 1.50	0.289
Missing	0.96	0.71 - 1.30	0.788
**Neutrophil: Lymphocyte Ratio**	1.01	1.00 - 1.01	0.153
**BMI**			
18.5-25 kg/m^2	Ref		
<18.5kg/m^2	1.28	0.72 - 2.27	0.404
25-30kg/m^2	0.92	0.66 - 1.29	0.643
>30kg/m^2	1.15	0.81 - 1.61	0.434
Missing	1.05	0.79 - 1.39	0.733
**eGFR (mL/min/1.73 m^2)**			
>90	Ref		
60-89	0.74	0.53 - 1.04	0.079
45-59	0.71	0.49 - 1.02	0.066
30-44	0.89	0.62 - 1.29	0.547
15-29	0.88	0.58 - 1.31	0.520

< 15	0.76	0.46 - 1.26	0.292
Missing	0.59	0.26 - 1.37	0.221
**Comorbidities**			
Diabetes Mellitus	1.05	0.83 - 1.31	0.693
Cardiovascular Disease	1.29	1.03 - 1.62	0.027
Respiratory Disease	1.14	0.91 - 1.43	0.255
Cancer	0.88	0.64 - 1.22	0.451
Mental Health	1.12	0.78 - 1.60	0.555
Dementia	1.22	0.93 - 1.60	0.142

Table S11 – Ordinal logistic regression for transitions of care using models 1 and 2

	Model 1			Model 2		
	OR	95% CI	P-value	OR	95% CI	P-value
**Frailty Distribution**						
1-3				Ref		
4				2.12	1.63 - 2.77	<0.001
5				2.85	2.19 - 3.70	<0.001
6				5.80	4.53 - 7.43	<0.001
7				7.74	5.93 - 10.11	<0.001
8				7.22	4.61 - 11.30	<0.001
9				5.59	2.13 - 14.69	<0.001
Missing				4.15	3.16 - 5.46	<0.001
**Age Distribution**						
18- 49 years	Ref			Ref		
50-64 years	1.74	1.34 - 2.25	<0.001	1.50	1.15 - 1.96	0.003
65-80 years	4.44	3.44 - 5.72	<0.001	2.55	1.95 - 3.35	<0.001
> 80 years	8.12	6.20 - 10.64	<0.001	3.54	2.64 - 4.75	<0.001
**Sex**						
Female	Ref			Ref		
Male	0.92	0.80 - 1.07	0.282	0.99	0.86 - 1.15	0.945
**NEWS**						
0-4 (Low risk)	Ref			Ref		
5-6 (Medium risk)	0.93	0.78 - 1.12	0.452	1.02	0.85 - 1.23	0.811
≥7 (High risk)	1.12	0.94 - 1.34	0.205	1.10	0.91 - 1.31	0.326
Missing	0.92	0.71 - 1.20	0.539	0.75	0.57 - 0.98	0.038
**CRP**						
< 10mg/l	Ref			Ref		
10-40mg/l	1.08	0.85 - 1.37	0.530	1.02	0.80 - 1.30	0.895
>40mg/l	1.18	0.95 - 1.47	0.130	1.18	0.94 - 1.47	0.148
Missing	2.48	1.71 - 3.59	<0.001	2.75	1.89 - 4.00	<0.001
**Ferritin**						
< 100 ng/ml	Ref			Ref		
100 – 1000 ng/ml	0.92	0.63 - 1.33	0.652	1.11	0.75 - 1.63	0.597
>1000 ng/ml	0.93	0.61 - 1.42	0.726	1.21	0.78 - 1.87	0.400
Missing	0.82	0.58 - 1.17	0.276	0.88	0.61 - 1.26	0.491
**Alanine transferase**						
< 40 IU/L	Ref			Ref		
> 40 IU/L	0.82	0.68 - 0.99	0.043	0.93	0.77 - 1.13	0.457
Missing	0.80	0.67 - 0.97	0.020	0.88	0.73 - 1.07	0.206
**Neutrophil: Lymphocyte Ratio**	1.00	1.00 - 1.01	0.413	1.00	0.99 - 1.00	0.444

**BMI**						
18.5-25 kg/m^2	Ref			Ref		
<18.5kg/m^2	1.50	1.03 - 2.17	0.033	1.32	0.90 - 1.93	0.151
25-30kg/m^2	1.11	0.90 - 1.36	0.324	1.28	1.04 - 1.57	0.021
>30kg/m^2	1.09	0.88 - 1.36	0.423	1.13	0.90 - 1.41	0.291
Missing	0.81	0.67 - 0.98	0.026	0.77	0.64 - 0.93	0.007
**eGFR (mL/min/1.73 m^2)**						
>90	Ref			Ref		
60-89	0.70	0.57 - 0.86	0.001	0.69	0.56 - 0.85	0.001
45-59	0.58	0.46 - 0.73	<0.001	0.61	0.48 - 0.77	<0.001
30-44	0.62	0.48 - 0.80	<0.001	0.64	0.49 - 0.83	0.001
15-29	0.88	0.65 - 1.19	0.406	0.83	0.62 - 1.13	0.245
< 15	1.36	0.94 - 1.96	0.098	1.18	0.82 - 1.71	0.373
Missing	1.60	1.06 - 2.40	0.024	1.63	1.08 - 2.45	0.020
**Comorbidities**						
Diabetes Mellitus	1.08	0.92 - 1.26	0.345	1.01	0.86 - 1.18	0.894
Cardiovascular Disease	0.88	0.76 - 1.02	0.081	0.85	0.73 - 0.99	0.037
Respiratory Disease	0.80	0.68 - 0.93	0.004	0.73	0.63 - 0.86	<0.001
Cancer	1.05	0.85 - 1.30	0.661	1.02	0.82 - 1.26	0.878
Mental Health	1.57	1.25 - 1.97	<0.001	1.29	1.02 - 1.62	0.033
Dementia	2.51	2.08 - 3.03	<0.001	1.48	1.21 - 1.81	<0.001
