# Supplementary material for: Age and frailty are independently associated with increased COVID-19 mortality and increased care needs in survivors: results of an international multi-centre study
Source: Age Ageing. 2021 Feb 5;50(3):617–30. doi: 10.1093/ageing/afab026 (PMC7929433; doi:10.1093/ageing/afab026)
Supplement: aa-20-1502-File004_afab026 [file aa-20-1502-file004_afab026.docx]

Age and frailty are independently associated with increased COVID-19 mortality and increased care needs in survivors: results of an international multi-centre study

**SUPPLEMENTARY DATA**

## Acknowledgements

Members of the Geriatric Medicine Research Collaborative:

**Writing and steering group**

| **Prefix** | **First name** | **Middle initial** | **Last name** | **Affiliation** |
| --- | --- | --- | --- | --- |
| Dr | Carly |  | Welch | University of Birmingham, UK |
| Dr | Daisy |  | Wilson |  |
| Dr | Lauren |  | McCluskey |  |
| Dr | Sarah |  | Richardson | AGE Research Group, NIHR Newcastle Biomedical Research Centre, University of Newcastle and Newcastle-upon-Tyne Hospitals NHS Foundation Trust, UK |
| Dr | Mary |  | Ni Lochlainn | King’s College London, UK |
| Dr | Jenni | K | Burton | University of Glasgow, UK |

**Advisory group**

| **Prefix** | **First name** | **Middle initial** | **Last name** | **Affiliation** |
| --- | --- | --- | --- | --- |
| Dr | Thomas | A | Jackson | University of Birmingham, UK |
| Dr | Krishnarajah |  | Nirantharakumar |  |
| Prof | Thomas |  | Pinkney |  |
| Dr | Laura |  | Magill |  |
| Prof | Miles |  | Witham | AGE Research Group, NIHR Newcastle Biomedical Research Centre, University of Newcastle and Newcastle-upon-Tyne Hospitals NHS Foundation Trust, UK |
| Dr | Claire |  | Steves | King’s College London, UK |
| Prof | Adam |  | Gordon | University of Nottingham, UK |
| Dr | Terence |  | Quinn | University of Glasgow, UK |

**Data analysis and data management**

| **Prefix** | **First name** | **Middle initial** | **Last name** | **Affiliation** |
| --- | --- | --- | --- | --- |
| Mr | Kelvin |  | Okoth | University of Birmingham, UK |
| Ms | Rita |  | Perry |  |
| Ms | Michala |  | Pettitt |  |
| Mr | Terry |  | Hughes |  |

**Regional and national representatives**

| **Prefix** | **First name** | **Middle initial** | **Last name** | **Affiliation** |
| --- | --- | --- | --- | --- |
| Dr | Muhammed |  | Elhadi | Alkhadra Hospital, Libya |
| Dr | Bryony |  | Brown | Bradford Teaching Hospitals NHS Foundation Trust, UK |
| Dr | Matthew |  | Hale |  |
| Dr | Benjamin |  | Jelley | Cardiff University, UK |
| Dr | Victoria |  | Gaunt | Gloucestershire Hospitals NHS Foundation Trust, UK |
| Dr | Melanie |  | Dani | Imperial College Healthcare NHS Trust, UK |
| Dr | Chee |  | Soo | Maidstone and Tunbridge Wells NHS Trust, UK |
| Dr | Isobel |  | Sleeman | NHS Grampian, UK |
| Dr | Mustafa |  | Alsahab | Oxford University Hospitals NHS Trust, UK |
| Dr | Katherine |  | Patterson | Queen's University Belfast, UK |
| Dr | Jennifer |  | Pigott | Royal Free London NHS Foundation Trust, UK |
| Dr | Adam |  | Seed | Southport and Ormskirk Hospital NHS Trust, UK |
| Dr | Kelli |  | Torsney | University of Cambridge, UK |
| Dr | Jane |  | Masoli | University of Exeter, UK |
| Dr | Elinor |  | Burn | University Hospitals of Leicester NHS Trust |
| Dr | Lucy |  | Beishon | University of Leicester, UK |
| Dr | Joanne |  | Taylor | University of Manchester, UK |
| Dr | Natalie |  | Cox | University of Southampton, UK |

**Protocol development and support**

| **Prefix** | **First name** | **Middle initial** | **Last name** | **Affiliation** |
| --- | --- | --- | --- | --- |
| Dr | Marie |  | Goujon | Cambridge University Hospitals NHS Foundation Trust, UK |
| Dr | Sarah |  | Freshwater | Health Education West Midlands, UK |
| Prof | Tahir |  | Masud | Nottingham University Hospitals NHS Trust, UK |
| Dr | Grace | ME | Pearson | University of Bristol, UK |
| Dr | Natalie |  | McNeela | Royal Wolverhampton NHS Trust, UK |
| Dr | Abigail |  | Reynolds |  |
| Dr | Asma |  | Khan | Sandwell and West Birmingham Hospitals NHS Trust, UK |
| Dr | Heena |  | Khiroya | University Hospitals Birmingham NHS Trust, UK |
| Dr | Katy |  | Madden |  |
| Dr | Nik |  | Sanyal |  |
| Dr | Awolkhier |  | Mohammedseid-Nurhussien | University Hospitals Coventry and Warwickshire NHS Trust, UK |
| Dr | Hannah |  | Moorey | University of Birmingham, UK |
| Dr | Christopher | N | Osuafor | University of Cambridge, UK |
| Dr | W | David | Strain | University of Exeter, UK |
| Dr | Rajni |  | Lal | Western Sydney Local Health District, Australia |

Members of the Covid Collaborative:

**Other collaborators**

| **First name** | **Middle initial** | **Last name** | **Affiliation** |
| --- | --- | --- | --- |
| Omar | A | Abdelwahab | Alazher University Hospital, Egypt |
| Elsayed | M | Awad |  |
| Ahmed | Y | Azzam |  |
| Almontacer | EB | Masood |  |
| Osama | MAS | Abdulhadi | Alkhadra Hospital, Libya |
| Hazem |  | Ahmed |  |
| Ahmed | KM | Hadreiez |  |
| Ahmed | A | Momen |  |
| Mosab | AA | Shaban |  |
| Hossam Aldein | S | Abd Elazeem | Assiut University Hospital, Egypt |
| Mohammed | H | Abdelhafez |  |
| Islam | A | Ahmed |  |
| Shrouk | M | Elghazaly |  |
| Helal | F | Hetta |  |
| Mohamed Eltaher | AA | Ibrahim |  |
| Soha | M | Mohamed |  |
| Aliae | AR | Mohamed Hussein |  |
| Mohamed | M | Moustafa |  |
| Mariam Albatoul |  | Nageh |  |
| Mahmoud | M | Saad |  |
| Alshaimaa | M | Saad |  |
| Omar |  | Zein Elabedeen |  |
| Victoria |  | Cox | Barnsley Hospital NHS Foundation Trust, UK |
| Danielle |  | Hunsley |  |
| Rebecca |  | Ryall |  |
| Kathleen | T | Shakespeare |  |
| Thyn |  | Thyn |  |
| Rachael |  | Webb |  |
| Deepthy |  | Hari Madhavan | Birmingham Heartlands Hospital, UK |
| Laxmi |  | Babar | City Hospital, Birmingham, UK |
| Tina |  | Doll |  |
| Agnieszka |  | Felska |  |
| Daniel | N | Guerero |  |
| Sandeep |  | Karthikeyan |  |
| Anne |  | Karunatilleke |  |
| Helena |  | Lee |  |
| Emma |  | Livesey |  |
| Amelia |  | Roberts |  |
| Charlotte |  | Roberts-Rhodes |  |
| Ahmed |  | Cordie | Cairo University Hospital, Egypt |
| Ahmed | O | Elmehrath |  |
| Mostafa |  | El-Shazly |  |
| Teresa |  | Perra | Cliniche San Pietro, A.O.U. Sassari, Italy |
| Alberto |  | Porcu |  |
| Antonio |  | Buondonno | Department of Medicine and Health Science "V. Tiberio", University of Molise, Italy |
| Giuseppe |  | Cecere |  |
| Aldo |  | Rocca |  |
| Vesna |  | Hogan | East Surrey Hospital, UK |
| Iain |  | Wilkinson |  |
| Ioannis |  | Baloyiannis | General University Hospital of Larissa, Greece |
| Jiannis |  | Hajiioannou |  |
| Konstantinos |  | Perivoliotis |  |
| George |  | Tzovaras |  |
| Anna |  | Fleck | Glasgow Royal Infirmary, UK |
| Aine |  | McGovern |  |
| Laura |  | Babb | Good Hope Hospital, UK |
| Emily |  | Bailey |  |
| Jay |  | Darley |  |
| Ioan | M | Draghita |  |
| Alexander |  | Hickman |  |
| Jason |  | Kalloo |  |
| Akhil |  | Kanzaria |  |
| Wasim |  | Nawaz |  |
| Ambreen |  | Sadiq |  |
| Rifa |  | Cardoso | Great Western Hospital, UK |
| Margherita |  | Faulkner |  |
| William |  | Hurst |  |
| Ellen |  | James |  |
| Aimee |  | Leadbetter |  |
| Jordan |  | Mayer |  |
| Tanya |  | Robinson |  |
| Emma |  | Stratton |  |
| Miriam |  | Thake |  |
| Hannah |  | Thould |  |
| Hannah |  | Watson |  |
| Sergio |  | Del Valle-Ruiz | Hospital General Reina Sofía, Spain |
| Nuria |  | Martínez-Sanz |  |
| Milagros |  | Carrasco-Prats |  |
| Pedro | V | Fernández- Fernández |  |
| Clara |  | Giménez-Francés |  |
| Esther |  | Medina-Manuel |  |
| Miguel |  | Ruiz-Marín |  |
| Pedro |  | López-Morales |  |
| Patricia |  | Pastor-Pérez |  |
| María |  | Valero-Soriano |  |
| Ismael |  | Mora-Guzmán | Hospital Santa Bárbara, Spain |
| Fabio |  | Barra | IRCCS Ospedale Policlinico San Martino, Italy |
| Antonella |  | Ferraiolo |  |
| Simone |  | Ferrero |  |
| Claudio |  | Gustavino |  |
| Chiara |  | Kratochwila |  |
| Eric | W | Etchill | Johns Hopkins Hospital, USA |
| Alodia |  | Gabre-Kidan |  |
| Joshua | H | Gray |  |
| Elliott | R | Haut |  |
| Harsha |  | Malapati |  |
| Sarah | F | Rapaport |  |
| Kent | A | Stevens |  |
| Dominique |  | Vervoort |  |
| Mohammed | A | Azab | King Abdullah Medical City Specialist Hospital, Saudi Arabia |
| Catherine |  | Bryant | King's College Hospital, UK |
| Hannah |  | Cheney-Lowe |  |
| Catrin |  | Cox |  |
| Andrew |  | Crowe |  |
| Gordon |  | Dick |  |
| Sarah |  | Evans |  |
| Patrick | CP | Hogan |  |
| Kar Yee |  | Law |  |
| Alexandra |  | Richardson |  |
| Fabio |  | Speranza |  |
| Kathryn |  | Toppley |  |
| Julie |  | Whitney |  |
| Eirene |  | Yeung |  |
| Alexandros |  | Charalabopoulos | Laiko University Hospital, Greece |
| Spyridon |  | Davakis |  |
| Amalia |  | Karapanou |  |
| Theodore |  | Liakakos |  |
| Efstratia |  | Baili |  |
| Maria |  | Mpoura |  |
| Michail | A | Sampanis |  |
| Nikolaos | V | Sipsas |  |
| Lucy |  | Beishon | Leicester Royal Infirmary, UK |
| Elinor |  | Burn |  |
| Parveen |  | Doddamani |  |
| Victoria |  | Haunton |  |
| Shahriar |  | Kabir |  |
| Hannah |  | Shaw |  |
| Chloe |  | Warner |  |
| Yasmin | K | NasrEldin | Minia University Hospital, Egypt |
| Nourhan | AA | Ghannam | Minya General Hospital, Egypt |
| Ravindra |  | Belgamwar | North Staffordshire Combined Healthcare NHS Trust, UK |
| Corrina |  | Bentley |  |
| Avinash |  | Aujayeb | Northumbria NHS Hospital Trust, UK |
| Lindsey |  | Dew |  |
| Catherine |  | Dotchin |  |
| James | M | Dundas |  |
| Elinor |  | Edwards |  |
| Georgia | F | Gilbert |  |
| Karl |  | Jackson |  |
| Sarah | H | Manning |  |
| Dominic |  | Maxfield |  |
| Nicholas |  | Moss |  |
| Declan | C | Murphy |  |
| Ellen |  | Tullo |  |
| Sarah | H | Welsh |  |
| Antonio |  | Buondonno | Policlinico San Pietro, Italy |
| Enrico |  | Pinotti |  |
| Francesco |  | Alessandri | Policlinico Umberto I, Sapienza University of Rome, Italy |
| Gioia |  | Brachini |  |
| Giancarlo |  | Ceccarelli |  |
| Flavia |  | Ciccarone |  |
| Pierfranco | M | Cicerchia |  |
| Bruno |  | Cirillo |  |
| Giorgio |  | De Toma |  |
| Giulia |  | Duranti |  |
| Enrico |  | Fiori |  |
| Giovanni | B | Fonsi |  |
| Pierfrancesco |  | Lapolla |  |
| Simona |  | Meneghini |  |
| Andrea |  | Mingoli |  |
| Francesco |  | Pugliese |  |
| Paolo |  | Sapienza |  |
| Luigi |  | Simonelli |  |
| Martina |  | Zambon |  |
| Caterina |  | Cattel | Princess Royal Hospital, King's College Hospital Trust, Surrey, UK |
| Laurenny |  | Guzman |  |
| Hannah |  | Dowell | Queen Alexandra Hospital, Portsmouth, UK |
| Aina |  | Ibukunoluwakitan |  |
| Fawsiya |  | Mohamed |  |
| Claire |  | Spice |  |
| Amanda |  | Stafford |  |
| Jolene |  | Atia | Queen Elizabeth Hospital Birmingham, UK |
| Catherine |  | Atkin |  |
| Hannah |  | Currie |  |
| Felicity |  | Evison |  |
| Zeinab |  | Majid |  |
| Maria |  | Qurashi |  |
| Siobhan |  | Coulter | Queen Elizabeth Hospital Gateshead, UK |
| Claire |  | McDonald |  |
| Georgina |  | Muir |  |
| Catherine |  | O'Mahony |  |
| Caroline |  | Tait |  |
| Rowan |  | Davies | Queen Elizabeth Hospital King's Lynn, UK |
| Katie |  | Honney |  |
| Laura |  | Winter |  |
| Olubayode |  | Adewole | Queen's Hospital Romford, UK |
| Amir |  | Abdelmalak | Queens Medical Centre, Nottingham, UK |
| Mohammed |  | Ahmad |  |
| Muhammed | H | Ansari |  |
| Kingsley |  | Appiah |  |
| Rajesh |  | Dwivedi |  |
| Hope |  | Elrick |  |
| Hedra |  | Ghobrial |  |
| Rosie |  | Jackson |  |
| Sophie |  | Jeffs |  |
| Sasha |  | Jeyakumar |  |
| Eleanor |  | Lunt |  |
| Bushra |  | Muzammil |  |
| Sylvia |  | Pytraczyk |  |
| Jonathan |  | Sheldrake |  |
| Jennifer |  | Smith |  |
| Hannah |  | Tobiss |  |
| Mark |  | Vettasseri |  |
| Ruth | H | Willott |  |
| Hein |  | Zaw |  |
| Moulinath |  | Bannerjee | Royal Bolton Hospital, UK |
| Jean |  | Cummings |  |
| Barbara |  | Hart |  |
| Tom |  | Maughan |  |
| Clare |  | Baguneid | Royal Derby Hospital, UK |
| Gabrielle |  | Budd |  |
| Lizzie |  | Moriarty |  |
| Omoteniola |  | Odutola |  |
| Hannah |  | Street |  |
| Alexis |  | Carr | Royal Devon and Exeter Hospital, UK |
| . |  | Royal Devon and Exeter Tissue Bank |  |
| Sarah |  | Baldwin | Royal Victoria Infirmary, UK |
| Hannah |  | Bashir |  |
| Jake |  | Gibbon |  |
| Amy |  | Gray |  |
| Grace |  | Lewis |  |
| Christina |  | Page |  |
| Rosanna |  | Varden |  |
| Anthony |  | Grubb | Royal Wolverhampton NHS Trust, UK |
| Elizabeth |  | Holmes |  |
| Harjinder |  | Kainth |  |
| Lara |  | Reilly |  |
| Mark |  | Whitsey |  |
| Mertcan |  | Akcay | Sakarya Faculty of Medicine, Turkey |
| Yeşim |  | Akdeniz |  |
| Emrah |  | Akın |  |
| Fatih |  | Altintoprak |  |
| Zülfü |  | Bayhan |  |
| Recayi |  | Capoglu |  |
| Hakan |  | Demir |  |
| Necattin |  | Firat |  |
| Emre |  | Gonullu |  |
| Tarık |  | Harmantepe |  |
| Baris |  | Mantoglu |  |
| Ali |  | Muhtaroglu |  |
| Merve |  | Yigit |  |
| Yasin | A | Yildiz |  |
| Lobna |  | Al-Sodani | Salford Royal Hospital, UK |
| Nicole |  | Burden |  |
| Evelyn |  | Charsley |  |
| Thomas |  | Kneen |  |
| Angeline |  | Price |  |
| Emma |  | Swinnerton |  |
| Yen Nee | J | Bo | Sandwell General Hospital, UK |
| Hayley | R | Boden |  |
| Reem |  | Bulla |  |
| Alison |  | Eastaugh |  |
| Helena |  | Lee |  |
| Mohammed |  | Mubin |  |
| Amelia |  | Roberts |  |
| Anthony |  | Umeadi |  |
| Stephanie |  | Wallis |  |
| Megan |  | Williamson |  |
| Yu Lelt |  | Win |  |
| Eltayeb | A | Ahmed | Sharq Alneel Hospital, Sudan |
| Abdulmoiz |  | Aljafari |  |
| Abdulmalek |  | Aljafari |  |
| Abdulkader |  | Mohammad |  |
| Ali |  | Ali | Sheffield Teaching Hospitals NHS Trust |
| Sylvia |  | Amini |  |
| James |  | Belcher |  |
| Marie |  | Giles |  |
| Hayley |  | Jarvis |  |
| Nathan |  | Jenko |  |
| Suvira |  | Madan |  |
| Alexander |  | Noar |  |
| Favour |  | Nwolu |  |
| Jessica |  | Parkin |  |
| Lauren | C | Passby |  |
| Jarita |  | Sivam |  |
| Michael |  | Surtees |  |
| Joanne |  | Wagland |  |
| Ruth |  | West |  |
| David |  | Williams |  |
| Manpreet |  | Badh | Solihull Hospital, UK |
| Amy |  | Birchenough |  |
| Nick |  | Coulthard |  |
| Alice |  | Devaney |  |
| Ratnam |  | Gandhi |  |
| Katharine |  | Hood |  |
| Samuel |  | North |  |
| Martha |  | Pinkney |  |
| Ellie |  | Shaw |  |
| Elisha |  | Whelan |  |
| Gurinder |  | Dogra | South Tyneside District Hospital, UK |
| Claire |  | Morris |  |
| Rebecca |  | Wright |  |
| Stephen |  | Lim | Southampton General Hospital, UK |
| Lia |  | Orlando |  |
| Harnish |  | Patel |  |
| Prabhleen |  | Puri |  |
| Sing Yang |  | Sim |  |
| Carolyn |  | Akladious | St Thomas' Hospital, UK |
| Gitanjali |  | Amaratungaz |  |
| Taha |  | Amir |  |
| Cheran |  | Anandarajah |  |
| Rachael |  | Anders |  |
| Sally |  | Aziz |  |
| Anna |  | Barnard |  |
| Monica |  | Bawor |  |
| Laura |  | Bremner |  |
| Hannah |  | Bridgwater |  |
| Hejab |  | Butt |  |
| Andra |  | Caracostea |  |
| Theodore |  | Chevallier |  |
| Victoria |  | Comerford |  |
| Jack |  | Cullen |  |
| Niamh |  | Cunningham |  |
| Daniel |  | Curley |  |
| Madeleine |  | Daly |  |
| Nikhita |  | Dattani |  |
| Benyamin |  | Deldar |  |
| Arjun |  | Desai |  |
| Nirali |  | Desai |  |
| Jugdeep |  | Dhesi |  |
| Maria |  | Dias |  |
| Hannah | C | Dooley |  |
| Samiullah |  | Dost |  |
| Hiren |  | Dusara |  |
| Alexander |  | Emery |  |
| Cassandra |  | Fairhead |  |
| Antia |  | Fernandez |  |
| Gracie |  | Fisk |  |
| Madeleine |  | Garner |  |
| Hannah |  | Gerretsen |  |
| Andrew |  | Ghobrial |  |
| Zaynub |  | Ghufoor |  |
| Deirdre |  | Green |  |
| Charlotte |  | Greene |  |
| Karla |  | Griffith |  |
| Ayushi |  | Gupta |  |
| Patrick |  | Harrison |  |
| Aidan |  | Haslam |  |
| Torben |  | Heinsohn |  |
| Lindsay |  | Hennah |  |
| Abigail |  | Hobill |  |
| Katherine |  | Hopkinson |  |
| Lara |  | Howells |  |
| Nicole |  | Hrouda |  |
| Irem |  | Ishlek |  |
| Rishi |  | Iyer |  |
| Nuha |  | Kardaman |  |
| Mairead |  | Kelly |  |
| Nicola | I | Kelly |  |
| Hesham |  | Khalid |  |
| Muhammad | S | Khan |  |
| Haris |  | Khan |  |
| Matthew |  | King |  |
| Li |  | Kok |  |
| Aneliya |  | Kuzeva |  |
| Rebecca |  | Lau |  |
| Gabriel |  | Lee |  |
| Gavriella |  | Levinson |  |
| Danielle |  | Lis |  |
| Baguiasri |  | Mandane |  |
| Jamie |  | Mawhinney |  |
| Henry |  | Maynard |  |
| Sophie |  | Mclachlan |  |
| Michelle |  | Metcalf |  |
| John |  | Millwood-Hargrave |  |
| Kelvin |  | Miu |  |
| Aaliya |  | Mohammed |  |
| Hamilton |  | Morrin |  |
| Stephanie |  | Mulhern |  |
| Daniel |  | Muller |  |
| Varun |  | Nadkarni |  |
| Hanna |  | Nguyen |  |
| Alice |  | O'Docherty |  |
| Sinead |  | O'Dwyer |  |
| Marc |  | Osterdahl |  |
| Ismini |  | Panayotidis |  |
| Shefali |  | Patel |  |
| Rose |  | Penfold |  |
| Rupini |  | Perinpanathan |  |
| Dina |  | Radenkovic |  |
| Thurkka |  | Rajeswaran |  |
| Tahmina |  | Razzak |  |
| Emily |  | Ross-Skinner |  |
| Hazel |  | Sanghvi |  |
| Ross |  | Sayers |  |
| Luca |  | Scott |  |
| Sri |  | Sivarajan |  |
| Katharine |  | Stambollouian |  |
| Jack |  | Stewart |  |
| Amybel |  | Taylor |  |
| Hrisheekesh |  | Vaidya |  |
| Vittoria |  | Vergani |  |
| Madiha |  | Virk |  |
| Vaishali |  | Vyas |  |
| Eleanor |  | Watkins |  |
| Catherine |  | Wilcock |  |
| Mettha |  | Wimalasundera |  |
| Stephanie |  | Worrall |  |
| Natalie |  | Yeo |  |
| Humza |  | Yusuf |  |
| Adam | H | Dyer | St. James's Hospital, Ireland |
| Cliona |  | Ni Cheallaigh |  |
| Liam |  | Townsend |  |
| Jocelyn |  | Amer | Sunderland Royal Hospital, UK |
| Emily |  | Lyon |  |
| Michael |  | Sen |  |
| Mohammed |  | Al-Sadawi | SUNY Downstate Brooklyn, USA |
| Adam |  | Budzikoski |  |
| Ishmam |  | Ibtida |  |
| Yusra |  | Qaiser |  |
| Mohammad | T | Azam | SUNY Upstate University Hospital, USA |
| Asad | J | Choudhry |  |
| William |  | Marx |  |
| Ahmad |  | Bouhuwaish | Tobruk Medical Center, Libya |
| Ahmed | SA | Taher |  |
| Nikolaos |  | Georgiou | Tunbridge Wells Hospital, UK |
| Jade |  | Man |  |
| Paul |  | Reynolds |  |
| Benjaman |  | To |  |
| Fatma | D | Collins | University Hospitals Coventry and Warwickshire NHS Trust, UK |
| Sharon |  | Budd |  |
| Ellanna |  | Griffin |  |
| Yue |  | Guan |  |
| Deevia |  | Hanji |  |
| Lily |  | Lowes |  |
| Farhana |  | Moomo |  |
| Olebu |  | Ogochukwu |  |
| Katie |  | Thin |  |
| Gilda |  | De Paola | University 'Magna Graecia' of Catanzaro, Italy |
| Gaetano |  | Gallo |  |
| Giuseppe |  | Sammarco |  |
| Giuseppina |  | Vescio |  |
| Shivam |  | Pancholi | University of Nicosia Medical School, Cyprus |
| Rand | A | Hussein | Zafaraniyah General Hospital, Iraq |
